# RAD23B Promotes Colorectal Cancer Metastasis via the Talin1/Integrin/PI3K/AKT/MMP9 Axis

**DOI:** 10.32604/or.2025.067535

**Published:** 2025-10-22

**Authors:** Jun Li, Yang Chen, Zhijiao Hao, Zhiyong Zhang, Jingyi Fan, Xiao Liu, Xueli Zhao, Hongyan Zhang, Chenpeng Wu

**Affiliations:** 1 Department of Clinical Laboratory, Tangshan Gongren Hospital, Tangshan, 064300, China; 2 Clinical School of North China University of Science and Technology, Tangshan, 064300, China; 3 Department of Pathology, Tangshan Gongren Hospital, Tangshan, 064300, China; 4 Department of Clinical Laboratory, Tangshan Rehabilitation Medical Center, Tangshan, 064300, China

**Keywords:** Colorectal cancer, radiation sensitive 23 homolog B, metastasis

## Abstract

**Background:**

Radiation sensitive 23 homolog B (RAD23B), a DNA repair-related protein, plays a contributory role in the development of multiple malignancies. This study aimed to explore the role of RAD23B in promoting colorectal cancer (CRC) metastasis and to elucidate the underlying molecular mechanisms.

**Methods:**

RAD23B was overexpressed in CRC cell lines SW480 and HCT-8, with empty vectors serving as controls. Invasion, cell proliferation, and migration were assessed using CCK-8 and Transwell assays. A xenograft mouse model was used to evaluate metastatic potential *in vivo*. Immunoprecipitation-mass spectrometry (IP-MS) and transcriptomic analysis by RNA sequencing (RNA-seq) were performed to identify signaling pathways regulated by RAD23B. Western blotting was used to analyze the expression of RAD23B, Talin1, Integrins αv/β1, PI3K, p-PI3K, AKT, p-AKT, and MMP9. Immunohistochemistry was conducted to examine RAD23B and Integrin β1 expression in CRC tissues.

**Results:**

RAD23B overexpression notably enhanced CRC migration, cell proliferation, and invasion both *in vitro* and *in vivo*. IP-MS, RNA-seq, and protein analysis revealed that RAD23B upregulated Talin1 and Integrins αv/β1, resulting in an activation of the PI3K/AKT signaling pathway. Moreover, RAD23B promoted MMP9 expression, contributing to enhanced invasive potential.

**Conclusion:**

RAD23B facilitates CRC metastasis through activation of the Talin1/Integrin αv/β1/PI3K/AKT/MMP9 signaling axis. These results provide novel insights into the role of RAD23B in CRC progression and identify it as a potential therapeutic target.

## 1 Introduction

Colorectal cancer (CRC) ranks among the foremost causes of cancer-related mortality globally, with its incidence and death rates steadily increasing, especially across developing nations [1]. According to the WHO, approximately 1.9 million new cases of CRC are diagnosed globally each year, resulting in around 900,000 deaths [2]. In China, the raising prevalence of CRC is closely linked to lifestyle changes and the Westernization of dietary habits [3]. Data from the National Cancer Center of China indicate that CRC stands as the second main cause of cancer-linked mortality after lung cancer, with death rates reaching 19.78 per 100,000 in men and 14.10 per 100,000 in women [4]. Despite advances in treatment strategies such as surgery and chemotherapy, therapeutic outcomes remain limited due to the frequent occurrence of metastasis [5]. Metastasis involves a series of complex biological processes, including tumor cell invasion, migration, and adaptation [6]. Thus, comprehending the molecular mechanisms that drive CRC metastasis is essential for improving early diagnosis, developing targeted therapies, and enhancing patient survival.

The mechanisms underlying CRC metastasis are multifactorial, involving epithelial-mesenchymal transition (EMT), genetic mutations, the emergence of metastasis-initiating cells (MICs), and the tumor microenvironment (TME) [7]. The Extracellular matrix (ECM), a dynamic component of the TME, plays a pivotal role in modulating tumor cell behavior and metastatic potential. As the structural scaffold of tissues, the ECM, along with its interactions with tumor cells, is a key determinant of metastatic capacity in CRC [8]. Talin1, a cytoskeletal protein, is critical for cell adhesion by linking the cell membrane to the ECM, thereby regulating migration, adhesion, and cell morphology [9]. Talin1 has also been closely associated with increased tumor invasiveness and metastatic progression [10]. Integrins, a widely expressed family of cell membrane adhesion receptors, mediate the mechanical linkage to the ECM and activate intracellular signaling cascades that govern tumorigenesis, progression, and metastasis [11]. Talin1 facilitates conformational changes in the extracellular domains of integrins, leading to their activation and enhanced binding affinity for ECM ligands. By activating signaling pathways such as phosphoinositide 3-kinase (PI3K)/protein kinase B (AKT) and modulating the expression of matrix metalloproteinases (MMPs), integrins regulate cell invasion and metastatic behavior [11–13]. In particular, MMP-9 degrades ECM components, promotes metastasis and tumor cell invasion, and serves as a potential diagnostic and prognostic biomarker [14,15].

Radiation sensitive 23 homolog B (RAD23B), a gene involved in DNA repair, cell cycle control, and tumorigenesis, has recently gained attention for its role in cancer metastasis [16]. It modulates several signaling pathways that influence cellular proliferation, migration, and invasion [17]. Previous studies conducted by our research group demonstrated that RAD23B promotes metastasis and CRC cell invasion [18]; however, its downstream molecular mechanisms and clinical significance remain incompletely understood. Thus, this study aimed to clarify the role of RAD23B in CRC metastasis and to explore the molecular mechanisms by which it regulates tumor cell migration, invasion, and metastasis through the Talin1/Integrin β1/PI3K/AKT/matrix metalloproteinase 9 (MMP9) signaling axis.

## 2 Materials and Methods

### 2.1 Cell Culture and Treatment

Human colorectal cancer cell lines SW480 and HCT-8 were obtained from the National Infrastructure of Cell Line Resource (Beijing, China). Cells were cultured in RPMI-1640 medium (VivaCell Biotechnology GmbH, Heidelberg, Germany) supplemented with 10% FBS and 1% streptomycin-penicillin (Hyclone; Cytiva, Logan, UT, USA), under standard conditions of 37°C in a humidified atmosphere with 5% CO_2_. The authenticity of each cell line was verified through short tandem repeat (STR) profiling, and all cultures were routinely screened to ensure the absence of mycoplasma contamination, maintaining experimental integrity throughout.

### 2.2 Cell Transfection and Transduction

Approximately 1 × 10^5^ SW480 and HCT-8 cells were plated into six-well plates, which were then allowed to adhere for 24 h before transfection. To achieve RAD23B overexpression, cells were transfected with either a pcDNA3.1-RAD23B construct or an empty pcDNA3.1 vector, both obtained from Charles River Laboratories (Wilmington, MA, USA), utilizing Lipofectamine™ 3000 (Thermo Fisher Scientific, Waltham, MA, USA; Cat. No. L3000001). Cells were subsequently divided into two groups: OE-NC (negative control) and OE-RAD23B (RAD23B-overexpressing).

### 2.3 Cell Counting Kit-8 (CCK-8) Assay

Roughly 5 × 10^3^ SW480 and HCT-8 cells were plated into 96-well plates, which were then incubated for 24 h. Following treatment, cells were cultured for 1 to 5 days, and their proliferation was assessed through the use of the CCK-8 assay kit (Cat. No. RP-RC3028; Hebei Ruipate Bio & Technology Co., Ltd., Shijiazhuang, China). In short, 10 μL of CCK-8 solution was dispensed slowly into each well, followed by a 2-h incubation period to allow for color development. The optical density was then recorded at 450 nm using a Multiskan™ FC Microplate Reader (Thermo Fisher Scientific, Waltham, MA, USA). Each experiment was independently repeated 3 times, and data are reported as the average of these replicates.

### 2.4 Transwell Assay

Cell migration and invasion assays were performed using Transwell chambers pre-coated with Matrigel^®^ (Corning Inc., Corning, NY, USA) to simulate the extracellular matrix. SW480 and HCT-8 cells were resuspended in serum-free RPMI-1640 medium at a concentration of 2 × 10^5^ cells/mL. After the designated treatments, 100 μL of the cell suspension was seeded into the upper compartment of each chamber, while the lower wells were filled with RPMI-1640 medium containing 10% fetal bovine serum (FBS) to serve as a chemoattractant. Chambers were incubated at 37°C for 48 h to allow for cellular migration and invasion. Post-incubation, non-migrated cells on the upper membrane surface were gently removed with a cotton swab. Cells that traversed to the lower surface were fixed in 10% paraformaldehyde, stained with 0.1% crystal violet, and examined under an inverted Nikon TE2000-S microscope (Tokyo, Japan). Quantification was performed by counting cells in five randomly selected microscopic fields per membrane.

### 2.5 Xenotransplant Murine Model

Approximately 1 × 10^5^ SW480 cells were plated into six-well plates and cultured for 24 h. When cell confluency reached around 60%, transfection was performed. The multiplicity of infection (MOI) was determined according to the protocol provided by Vigene Biosciences (Jinan, China), and lentiviral particles were added to reach a final concentration of 1 × 10^8^ U/mL. Following 24 h of incubation, the culture medium was refreshed, and puromycin (2 μg/mL) was introduced to initiate selection of successfully transduced cells. In parallel, an untreated control group was maintained to monitor selection efficiency. Once complete cell death was noted in the control group, the puromycin concentration was reduced to 1 μg/mL to sustain selective pressure. Upon reaching full confluence, the surviving resistant cells were harvested for downstream analyses.

For the *in vivo* metastasis model, four- to six-week-old NOD/ShiLtJGpt-Prkdc^em26Cd52^Il2rg^em26Cd22^/ Gpt (NCG) mice (GemPharmatech, Nanjing, China) were used. The mice were kept under SPF conditions in an environment with a temperature of 22 ± 2°C, humidity between 50% and 60%, and a 12-h light/dark cycle. They had free access to food and water throughout the experiment. Moreover, after a 1-week acclimatization period, SW480 cells stably overexpressing RAD23B (OE-RAD23B, *n* = 7) or carrying the OE-NC control plasmid (*n* = 7) were prepared in PBS (pH 7.4) at a concentration of 1 × 10^7^ cells/mL. Each mouse received a 50 μL injection of the cell suspension into the splenic tail region. Body weight was monitored every three days during the study.

Between 20 and 28 days after injection, the mice were anesthetized and humanely euthanized. Their livers were collected and inspected for the presence of metastatic nodules. The tissues were then fixed in 4% neutral-buffered formaldehyde, followed by routine paraffin embedding and sectioning for histopathological analysis. Liver sections were stained utilizing a H&E Staining Kit (Cat. No. C0105S, Beyotime Biotechnology, Beijing, China). The procedure involved staining with hematoxylin for 5 min, briefly rinsing with distilled water for 3 s to remove excess stain, and then bluing under running tap water for 10 min. After a quick rinse in distilled water (5 s), they were counterstained with eosin for 30 s. Dehydration and rehydration procedures were performed as in standard immunohistochemistry (IHC) protocols. Transverse sections (4 μm thick) were taken from five diverse regions of each liver to ensure comprehensive analysis. H&E staining was used to assess micrometastases, which were quantified and statistically analyzed in mice injected with either OE-RAD23B or OE-NC SW480 cells. Microscopic images were obtained using a Nikon TE2000-S microscope (Tokyo, Japan). All procedures involving animals were conducted per institutional guidelines and were approved by the Institutional Animal Care and Use Committee of North China University of Science and Technology (Approval No. SQ2022003).

### 2.6 Immunoprecipitation (IP)-Mass Spectrometry (MS)

For IP-MS analysis, 50 μL of magnetic beads (Cat. No. AM001-02; ACE Biotechnology, Nanjing, China) were transferred to 1.5 mL Eppendorf MicroTest tubes and placed on a magnetic rack (Invitrogen™; Thermo Fisher Scientific Inc.). After 1 min, the supernatant was discarded, and the beads were washed twice with 500 μL of 0.02% PBS-Tween 20. Subsequently, RAD23B antibody (2 μg/mL; Cat. No. ab86781, Abcam, Cambridge, MA, USA), prepared by diluting a 0.2 mg/mL stock at a 1:100 ratio, was added to the magnetic beads. The mixture was gently rotated and incubated for 2 h at room temperature. Following incubation, the beads were washed twice with 0.02% PBS containing Tween 20.

To facilitate antibody crosslinking, two sequential washes were performed with 2 M triethanolamine (pH 8.2) to cleanse the beads. Then, 1 mL of 20 mM immediately prepared dimethyl pimelimidate (DMP) in 0.2 M triethanolamine was gently mixed in and left to incubate at room temperature for 30 min. After discarding the supernatant, 1 mL of 50 mM Tris buffer (pH 7.5) was added, and the beads were resuspended gently with a pipette. The tubes were placed on a rotating shaker (model ZWF-334; ZHCHENG, Shanghai, China) at 30 rpm for 1 min to terminate the crosslinking reaction. The beads were then washed twice more with 0.02% PBS-Tween 20.

For protein extraction, 1 mL of lysate sample was maintained at 4°C for 2 h under gentle rotation with the magnetic bead complex, followed by three washes with 1% NP-40 buffer (pH 7.4; Cat. no. P0013F, Beyotime Biotechnology, Beijing, China). The final samples were submitted to PTM BIO (Jingjie Biotechnology Co., Ltd., Hangzhou, China) for LC-MS/MS analysis. IP-MS was performed using SW480 cells. The raw IP-MS data have been deposited in the ProteomeXchange database under project accession number PXD062847 (Reviewer Token: 9oakbEZGEKwb). Volcano plots were generated utilizing the R package ‘ggplot2’ (3.4.4) to visualize differentially expressed proteins based on fold change and adjusted *p*-values.

### 2.7 Transcriptome Analysis

Total RNA was extracted from SW480 cells in both the OE-RAD23B and OE-NC groups utilizing TRIzol™ reagent (Invitrogen; Thermo Fisher Scientific Inc.; Cat. No. 15596026). RNA purity was assessed by measuring the A260/A280 ratio, with values between 1.8 and 2.0 considered acceptable. RNA integrity was evaluated via gel electrophoresis. Only high-quality RNA samples were used to construct RNA sequencing (RNA-seq) libraries using a standard library preparation kit. The libraries were sequenced on the Illumina NovaSeq 6000 platform (Novogene Co., Ltd., Beijing, China). Differentially expressed genes (DEGs) were identified using DESeq2 software [19] (https://github.com/mikelove/DESeq2, version 1.16.1, accessed on 20 July 2025), with thresholds set at |log_2_FoldChange| > 1 and *p* < 0.05. DEGs were subjected to functional enrichment analysis via the ‘ClusterProfiler’ (4.6.2) package [20] (https://bioconductor.org/packages/clusterProfiler/, version 3.4.4, accessed on 20 July 2025) for Gene Ontology (GO) and Kyoto Encyclopedia of Genes and Genomes (KEGG) pathway analyses. The RNA-seq raw data are available for download at: https://www.ncbi.nlm.nih.gov/sra/PRJNA1247928 (accessed on 20 July 2025). Gene Set Enrichment Analysis (GSEA) was performed utilizing the ‘ClusterProfiler’ package to identify significantly enriched pathways based on the DEGs.

### 2.8 Toxicogenomics Database Analysis

To identify potential compounds that may negatively regulate RAD23B expression, we utilized the GeneCards database, which integrates gene-related data from multiple sources, including the Comparative Toxicogenomics Database (CTD). The CTD provides curated information on chemical-gene-disease interactions.

### 2.9 Western Blot

Proteins were isolated from SW480 and HCT-8 cells utilizing RIPA lysis buffer (BIOSS) supplemented with protease inhibitors. The protein concentration was measured using a BCA assay kit (Jiangsu Kaiji Biotechnology Co., Ltd., Nanjing, China; cat. no. KGB2101-500). Equal amounts of protein (30 μg per sample) were then subjected to SDS-PAGE using a 10% polyacrylamide gel and subsequently transferred onto PVDF membranes.

The membranes were blocked with 10% skim milk in PBST for 1 h at room temperature. PBST was prepared by diluting 10× TBST buffer (cat. no. ZS405-3; ZOMANBIO, Beijing, China), which contains 200 mM Tris-HCl, 1.5 M NaCl, and 1% Tween-20 (pH 7.6), with distilled water. After blocking, an overnight incubation at 4°C was carried out with the designated primary antibodies. All diluted 1:1000 unless otherwise stated: RAD23B (cat. no. A1034; ABclonal Biotech Co., Ltd., Wuhan, China); Talin1 (cat. no. 14168-1-AP; Proteintech Group Inc., Rosemont, IL, USA); Integrin αv (cat. no. 4711S; Cell Signaling Technology Inc., Danvers, MA, USA); Integrin β1 (cat. no. 34971S; Cell Signaling Technology Inc.); Phosphorylated PI3K (p-PI3K; cat. no. HA721672; Huaan Biotechnology Co., Ltd., Hangzhou, China); Total PI3K (cat. no. ET1608-70; Huaan Biotechnology Co., Ltd.); Phosphorylated AKT (p-AKT; cat. no. ET1607-73; Huaan Biotechnology Co., Ltd.); Total AKT (cat. no. HA721870; Huaan Biotechnology Co., Ltd.); MMP9 (cat. no. ET1704-69; Huaan Biotechnology Co., Ltd.); β-actin (1:3000; cat. no. AC026; ABclonal Biotech Co., Ltd.)

Following three washes with PBST, membranes were incubated at room temperature with goat anti-rabbit IgG secondary antibodies (1:5000; cat. no. S1002-100; Hebei Ruipate Bio & Technology Co., Ltd., Shijiazhuang, China). Protein bands were visualized using the ChemiScope 6000 gel imaging system (Clinx Science Instruments Co., Ltd., Shanghai, China) and detected with the SuperECL Plus chemiluminescence kit (cat. no. P1050; Applygen Technologies Inc., Beijing, China).

### 2.10 IHC

A tissue microarray comprising 90 CRC samples and 90 matched adjacent non-cancerous tissues was obtained from Shanghai Outdo Biotech Co., Ltd. (Shanghai, China, Cat. No. HRec-Ade180Sur-03) (Table 1). The patients included ranged in age from 31 to 90 years, with a median age of 63. Complete follow-up information was available for all individuals, with durations spanning 1 to 108 months.

**Table 1:** Correlation between integrin β1 expression and clinicopathological parameters

Clinicopathological parameters	*N* (90)	Integrin **β**1		*X* ^**2**^	*p*
		Low expression (*n*)	High expression (*n*)		
Sex					
Male	58	32	26	0.569	0.451
Female	32	15	17		
Age					
≥60	54	25	29	1.900	0.168
<60	36	22	14		
Tumor diameter (cm)					
≥7	18	13	5	3.607	0.058
<7	72	34	38		
T stage					
T3 + T4	77	41	36	0.224	0.636
T1 + T2	13	6	7		
N stage					
N1-2	43	17	26	5.312	0.021
N0	47	30	17		
M stage					
M1	4	1	3		0.345*
M0	86	46	40		
AJCC stage					
III + IV	44	18	26	4.416	0.036
I + II	46	29	17		
Vascular invasion					
Yes	5	1	4		0.189*
No	85	46	39		

Note: *Fisher’s exact test; AJCC, American Joint Committee on Cancer.

Tissue sections were sequentially deparaffinized and rehydrated via a graded ethanol series, which were then followed by a 10-min treatment with 0.3% hydrogen peroxide in methanol to quench endogenous peroxidase activity. Antigen retrieval was conducted by heating slides at 100°C for 30 min in 10 mM sodium citrate buffer (pH 6.0). After cooling to room temperature, sections were incubated overnight at 4°C with primary antibodies against RAD23B (1:50, Cat. No. A1034; ABclonal Biotech Co., Ltd.) and Integrin β1 (1:1000, Cat. No. 34971S; Cell Signaling Technology Inc.) [21]. Signal detection was performed using the Prolink-2 Plus HRP Rabbit Polymer Detection Kit (Cat. No. PV-6001; OriGene Technologies Inc., Rockville, MD, USA) per the manufacturer’s instructions. Digital slide imaging was completed using Aperio ScanScope software (version 8.0; Leica Biosystems, Nussloch, Germany).

Immunohistochemical staining was semi-quantitatively assessed based on the percentage of stained cells and staining intensity [22]. Two pathologists, blinded to clinical information, independently scored all sections. Integrin β1 expression intensity was rated as: 0 = negative, 1+ = weak (yellow), 2+ = moderate (light brown), and 3+ = strong (dark brown). The positive area was recorded as a percentage (0%–100%), and the final IHC score was obtained by multiplying the intensity score by the percentage of positive cells. Box plots were generated using R software to visualize group differences. The boxes represent interquartile ranges, with medians shown as horizontal lines. Statistical comparisons were conducted utilizing the Wilcoxon rank-sum test, and significance was marked where *p* < 0.05. The study received ethical approval from the Tangshan Gongren Hospital Ethics Committee (Approval No. GRYY-LL-2017-10).

### 2.11 Statistical Analysis

GraphPad Prism version 8.0 (GraphPad Software, San Diego, CA, USA) was utilzied for all statistical computations, and values are reported as mean ± standard deviation. Unpaired *t*-tests were used to compare means between two groups. Pearson’s correlation was applied to assess relationships between continuous variables. For data not following a normal distribution, non-parametric tests were employed to compare two independent groups. Categorical data were analyzed using either the Chi-square test (χ^2^) or Fisher’s exact test, depending on the context. Kaplan–Meier survival analysis was used to assess patient outcomes, while univariate and multivariate Cox regression models were utilized to identify potential prognostic indicators in colorectal cancer. Hazard ratios (HRs) and 95% confidence intervals (CIs) were calculated utilizing the Cox models. All statistical analyses were performed utilizing R software (4.3.1). Differences were considered statistically significant at *p* < 0.05.

## 3 Results

### 3.1 RAD23B Promotes the Invasion, Proliferation, and Migration of CRC Cells In Vitro

**Figure 1:**
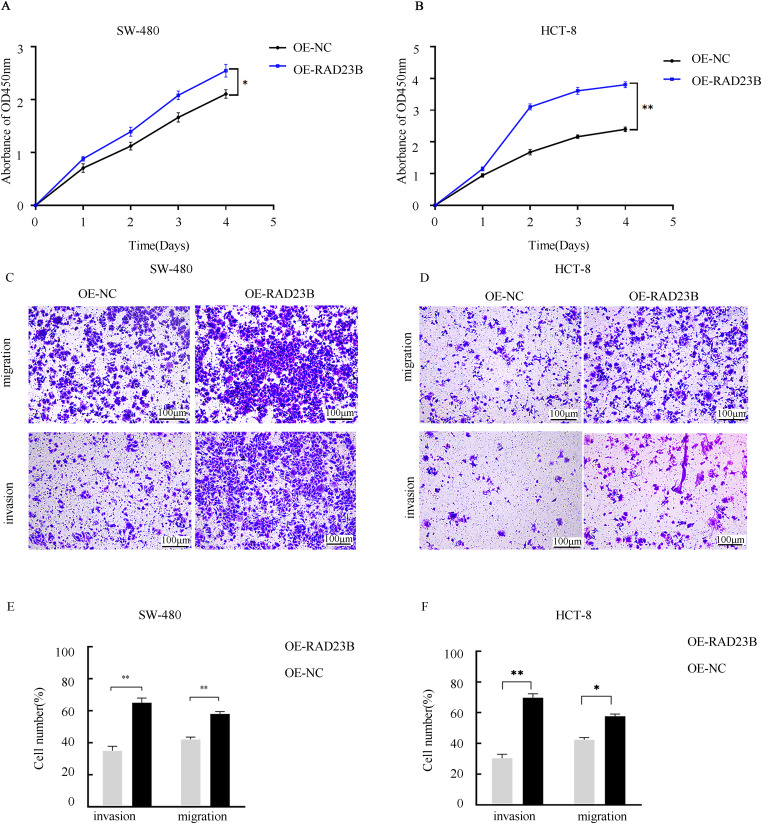
RAD23B overexpression promotes proliferation, migration, and invasion of CRC cells. CCK-8 assays were used to assess the proliferation of (**A**) SW480 and (**B**) HCT8 CRC cells overexpressing RAD23B compared with control cells. Absorbance at 450 nm was measured at various time points. Transwell assays were conducted to evaluate the migration and invasion abilities of (**C**) SW480 and (**D**) HCT8 cells overexpressing RAD23B. Representative images are shown; scale bars, 100 μm. Quantification of migrated and invaded cells in (**E**) SW480 and (**F**) HCT8 cells is presented. Each experiment was performed in triplicate and repeated on at least three separate occasions. Data are reported as mean ± SD. *p < 0.05; **p < 0.01

To investigate the role of RAD23B in CRC cell proliferation, SW480 and HCT-8 cells were transfected with either an OE-RAD23B plasmid or an OE-NC plasmid. Cell viability was measured over five days using the CCK-8 assay. Cells overexpressing RAD23B exhibited significantly higher proliferation rates than the control group on days 1, 2, 3, 4, and 5 (Fig. 1A, *p* < 0.05; Fig. 1B, *p* < 0.01), indicating enhanced proliferative capacity. To evaluate the impact of RAD23B on cell motility, Transwell assays were performed to assess invasion and migration. RAD23B-overexpressing SW480 and HCT-8 cells showed significantly enhanced invasion and migration relative to the control group. In Fig. 1C,E, both migration and invasion were markedly enhanced (*p* < 0.01). In Fig. 1D,F, invasion was significantly enhanced (*p* < 0.01), while migration also showed a moderate but statistically significant elevation (*p* < 0.05). These findings imply that RAD23B promotes CRC migration, cell proliferation, and invasion.

### 3.2 Overexpression of RAD23B Promotes CRC Metastasis In Vivo

**Figure 2:**
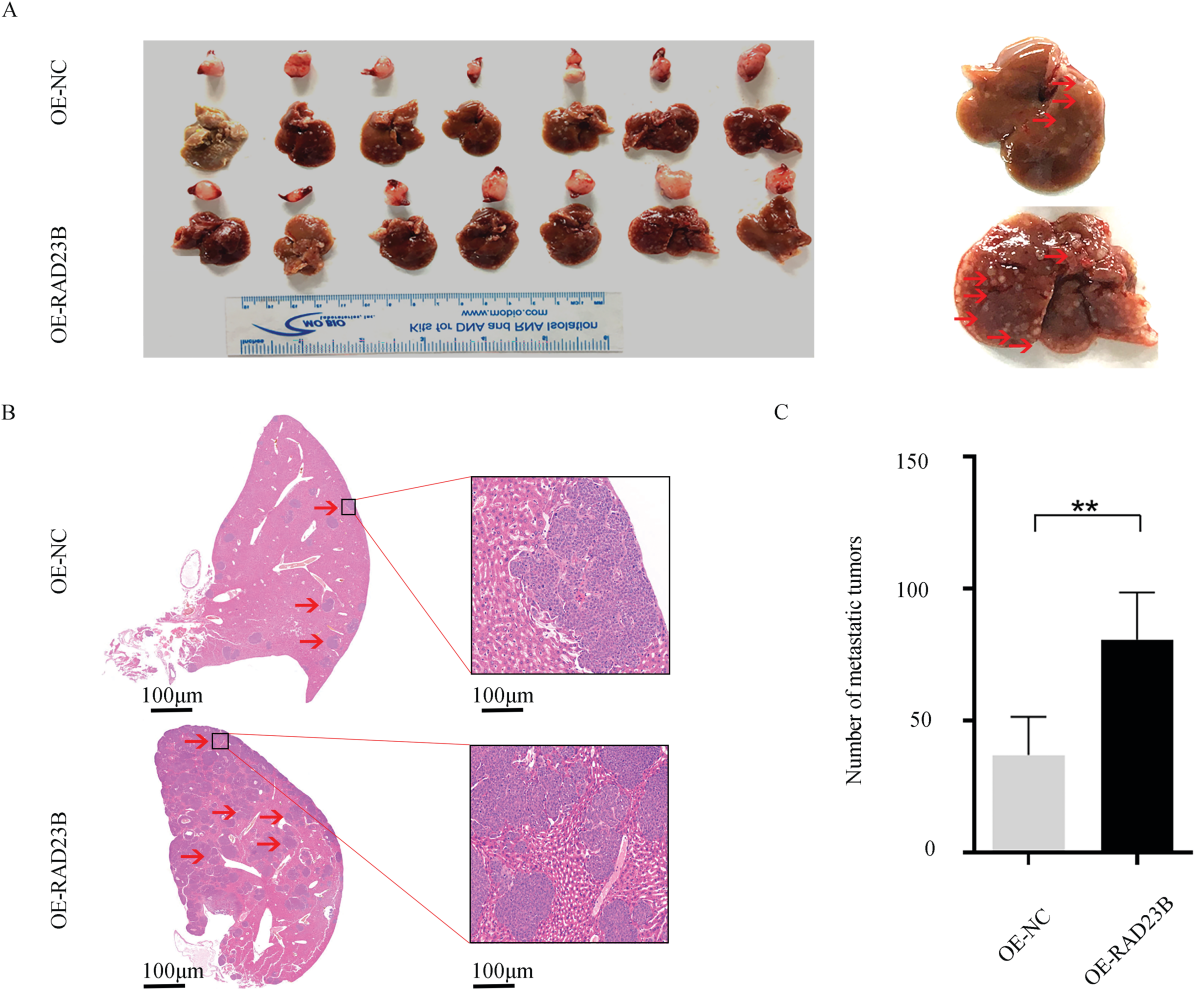
RAD23B overexpression promotes CRC metastasis *in vivo*. (**A**) Representative images of liver and spleen tissues from mice orthotopically injected in the spleen with SW480 cells stably overexpressing RAD23B or control cells. (**B**) H&E staining of liver metastatic tumors from the two groups. (**C**) Quantification of liver tumor metastases derived from the two groups. **p < 0.01

To evaluate whether RAD23B overexpression enhances the metastatic potential of CRC cells *in vivo*, SW480 cells stably overexpressing RAD23B or a control plasmid were generated. Fourteen NCG mice (*n* = 14) were randomly grouped into two cohorts, revealing a pronounced elevation in liver metastasis incidence in the RAD23B overexpression group vs. the control. (*p* < 0.01; Fig. 2). These findings indicate that RAD23B overexpression promotes the metastatic capacity of CRC cells *in vivo*.

### 3.3 IP-MS Reveals RAD23B-Associated Proteins

**Figure 3:**
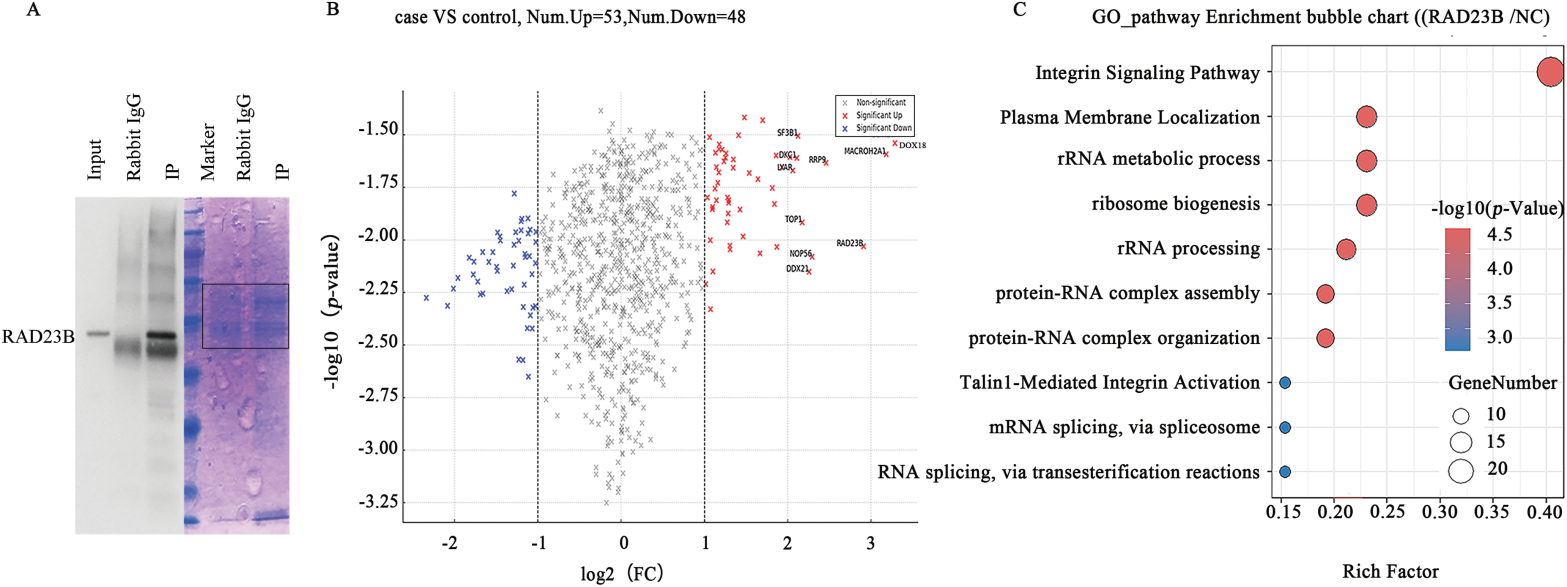
Identification of RAD23B-interacting proteins by IP-MS. (**A**) Coomassie blue staining of immunoprecipitated proteins using RAD23B antibodies (IP) and IgG controls. The red box indicates the region subjected to liquid chromatography-MS/MS analysis. (**B**) Volcano plot illustrating differentially expressed proteins in RAD23B IP vs. IgG control. Significantly enriched proteins (*p* < 0.05; fold change > 2) are illustrated in red; significantly depleted proteins (*p* < 0.05; fold change < 0.5) are in blue. (**C**) GO and pathway enrichment analysis of RAD23B-interacting proteins identified by IP-MS. Top pathways are ranked by enrichment factor; point size reflects the number of genes, and color gradient indicates the adjusted *p-*value. IP, immunoprecipitation; MS, mass spectrometry; FC, fold change

IP-MS analysis identified RAD23B-associated proteins, revealing a significant enrichment of Talin1 and Integrin in RAD23B immunoprecipitates compared with IgG controls (Fig. 3A). Quantitative analysis further confirmed the interaction between RAD23B and both Talin1 and Integrin in SW480 cells (Fig. 3B,C), suggesting that RAD23B may promote metastasis through its association with these key adhesion molecules. The top RAD23B-interacting proteins identified by IP-MS are listed in Supplementary Table S1.

### 3.4 Transcriptome Analysis Identifies RAD23B-Regulated Pathways

**Figure 4:**
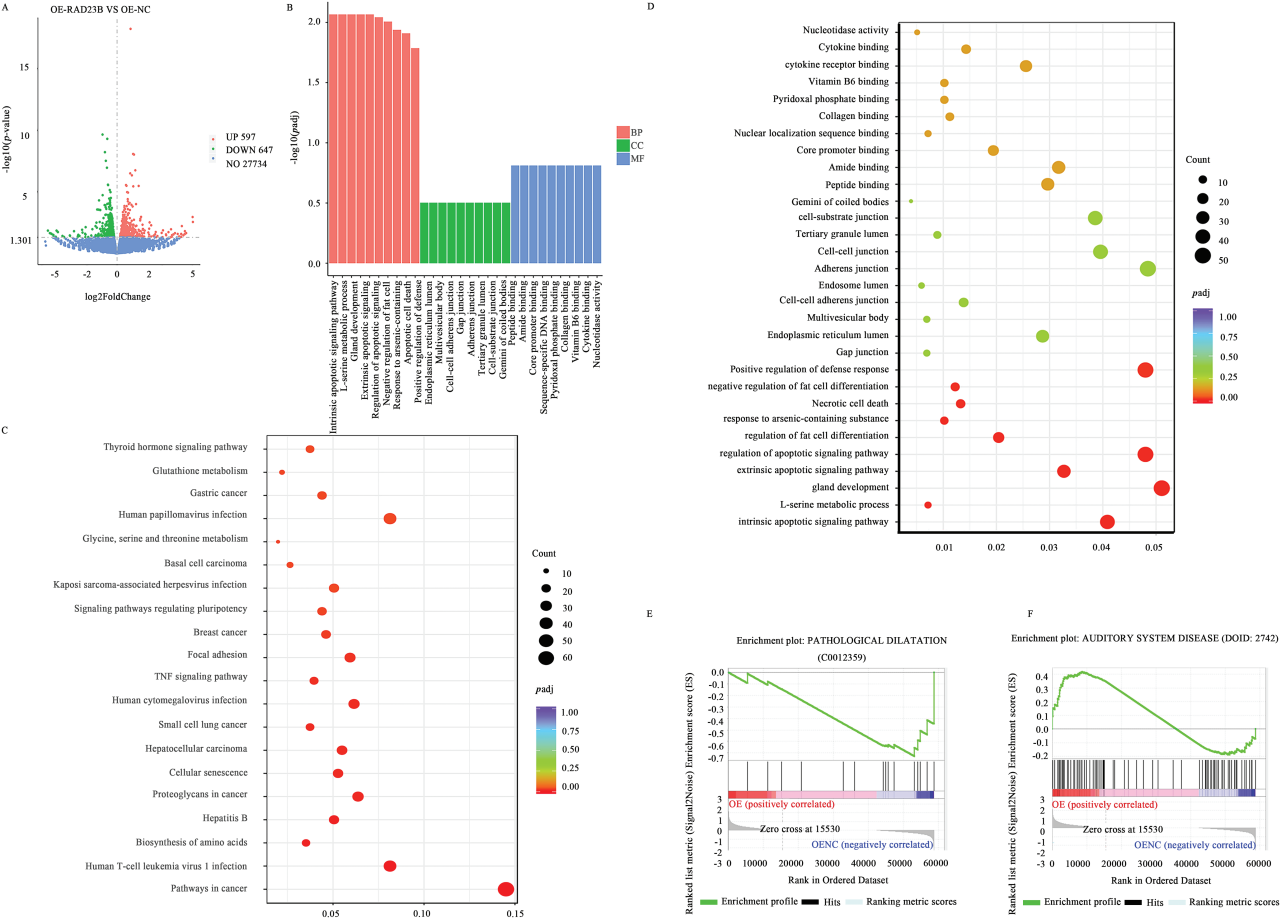
Transcriptomic analysis of downstream gene expression in CRC cells overexpressing RAD23B. (**A**) Volcano plot showing differential gene expression in SW480 cells overexpressing RAD23B compared with control cells. A total of 547 genes were upregulated and 667 genes were downregulated (*p* < 0.05; |log_2_FoldChange| >1). (**B**) GO enrichment analysis of DEGs. (**C**) KEGG pathway analysis highlighting Talin and MMP9 as notably related with RAD23B overexpression. (**D**) GO enrichment analysis showing pathways related to cell adhesion, ECM, and apoptosis regulation. (**E**) GSEA showing activation of the “PATHOLOGICAL DILATATION” pathway. (**F**) GSEA indicates enrichment of the “AUDITORY SYSTEM DISEASE” pathway

Transcriptome sequencing of SW480 cells with OE-RAD23B identified 547 upregulated and 667 downregulated genes (|log_2_FoldChange| > 1; *p* < 0.05; Fig. 4A). The top 100 upregulated and top 100 downregulated DEGs are listed in Supplementary Tables S2 and S3, respectively, with corresponding heatmaps shown in Supplementary Figs. S1 and S2. GO analysis revealed significant enrichment in pathways related to cell adhesion, ECM remodeling, and signal transduction (Fig. 4B–D). KEGG pathway analysis highlighted prominent activation of the PI3K/AKT signaling pathway, including upregulation of PI3K, AKT, and MMP9 (Fig. 4C). Furthermore, GSEA indicated activation of MMP9-associated pathways (Fig. 4E,F), supporting the role of RAD23B in promoting CRC metastasis through the Talin1/Integrin/PI3K/AKT/MMP9 signaling axis.

### 3.5 RAD23B Regulates the Talin1/Integrin/PI3K/AKT/MMP9 Axis

**Figure 5:**
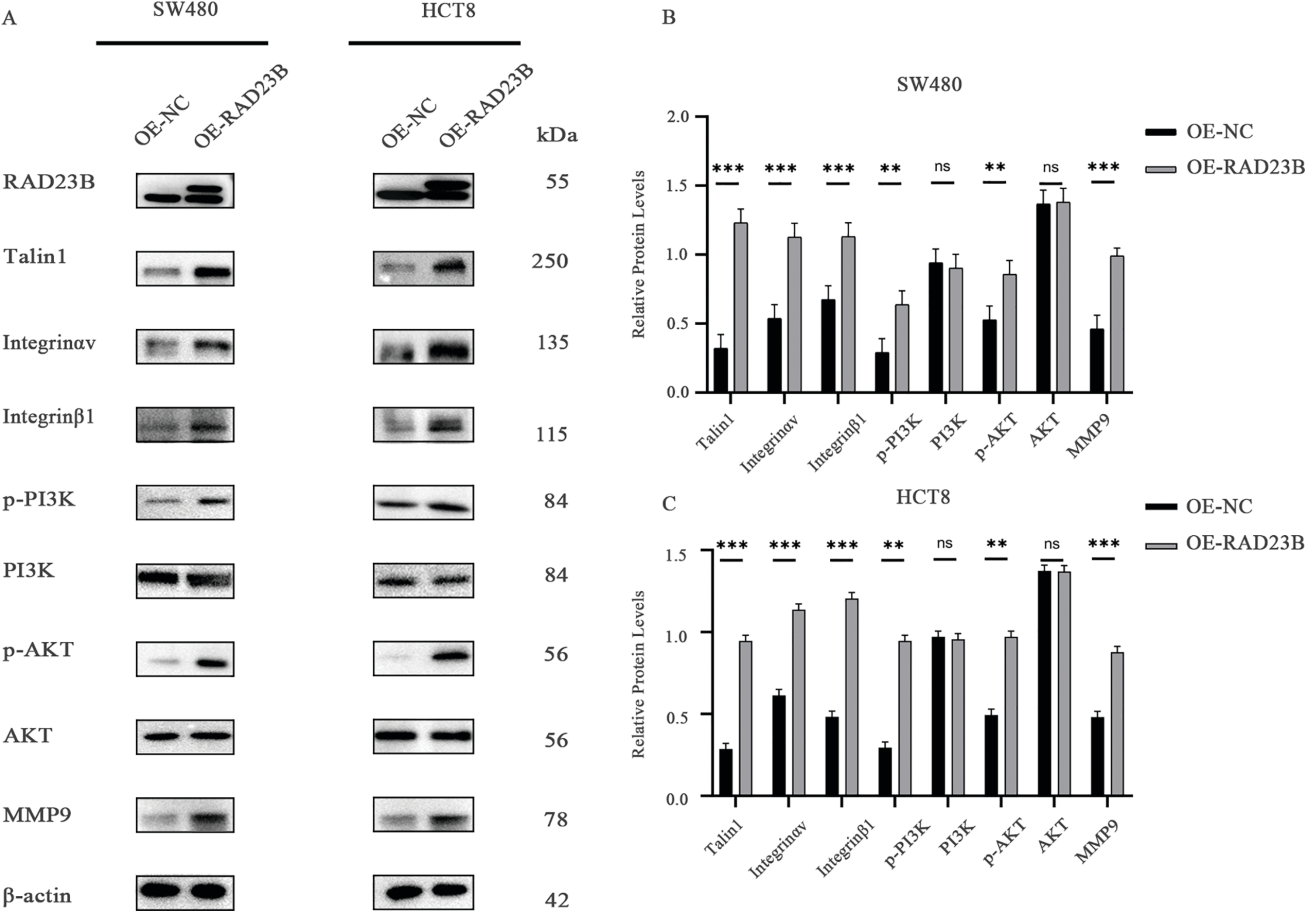
RAD23B activates the Talin-Integrin/PI3K/AKT signaling axis in CRC cells. (**A**) Western blot analysis of key proteins involved in the Talin-Integrin/PI3K/AKT pathway in HCT8 and SW480 cells overexpressing RAD23B. (**B**,**C**) Semi-quantification of protein expression levels in SW480 (**B**) and HCT8 (**C**) cells. ns, *p* > 0.05; **p < 0.01; ***p < 0.001

Western blot analysis of key signaling molecules in OE-RAD23B cells revealed increased expression of MMP9, Integrin β1, Integrin αv, and Talin1 compared with the OE-NC group (Figs. 5A–C, S3 and S4). Additionally, the levels of p-PI3K and p-AKT were significantly elevated in the OE-RAD23B group, indicating activation of the PI3K/AKT signaling pathway. These findings suggest that RAD23B promotes CRC metastasis by upregulating Talin1, Integrin, and MMP9 expression, in conjunction with activating the PI3K/AKT pathway.

### 3.6 RAD23B Expression is Correlated with Integrin ***β***1 in CRC Tissues

**Figure 6:**
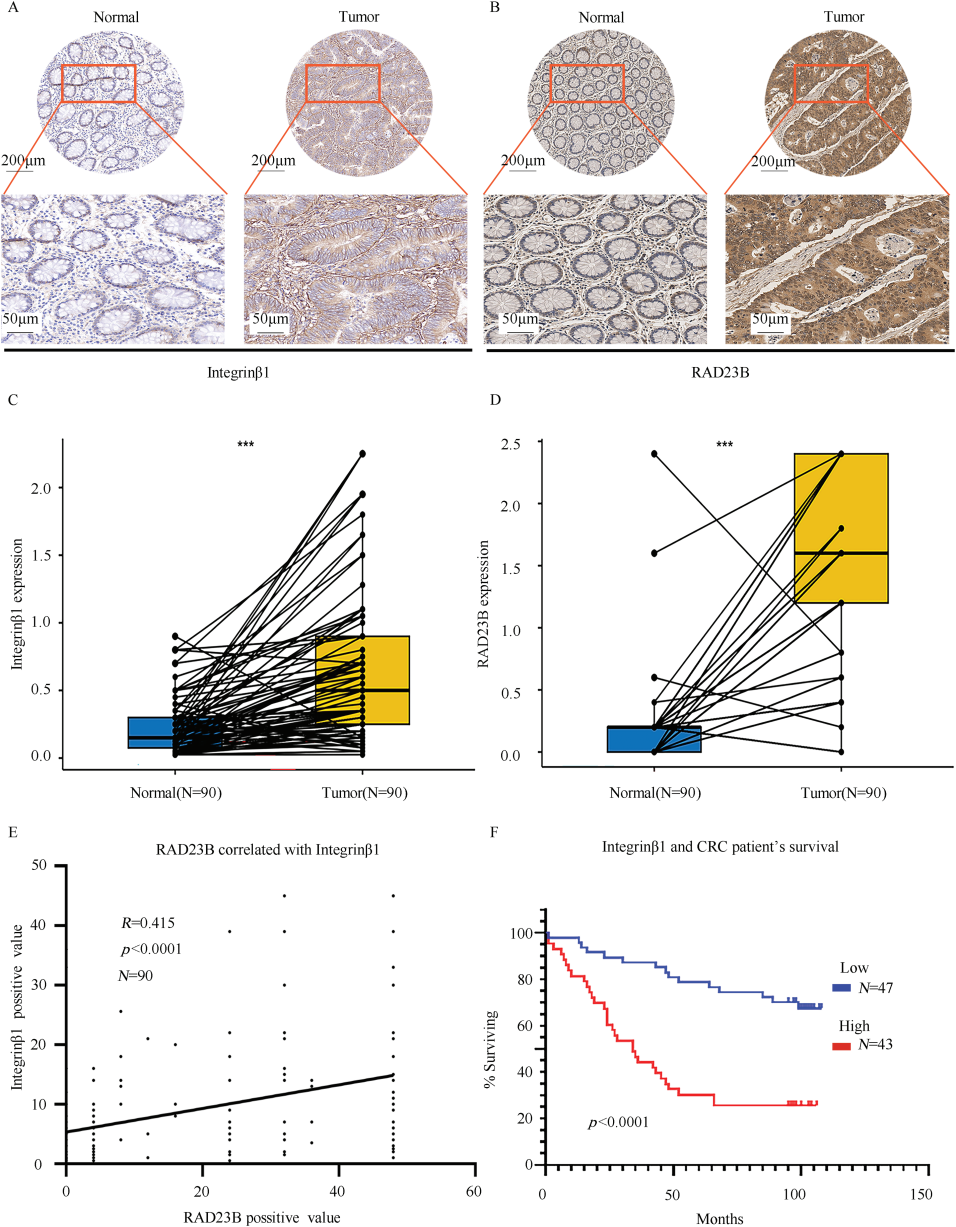
RAD23B and Integrin β1 are highly expressed in CRC tissues and are linked with poor patient survival. IHC analysis of (**A**) Integrin β1 and (**B**) RAD23B expression in CRC tumor tissues and paired adjacent normal tissues. Representative images are shown, with stronger staining observed in tumor tissues. Scale bars: 200 μm (upper panels) and 50 μm (lower panels). (**C**,**D**) Box plots showing significantly higher expression of Integrin β1 (**C**) and RAD23B (**D**) in CRC tissues (*n* = 90) compared to adjacent normal tissues. (**E**) Correlation analysis between RAD23B expression and the Integrin β1-positive area in CRC tissues (*n* = 90). (**F**) Kaplan-Meier survival curves showing that high Integrin β1 expression (*n* = 43) correlates with poorer overall survival compared with low expression (*n* = 47). ***p < 0.001

IHC analysis of CRC and adjacent normal tissues revealed significantly higher expression levels of both RAD23B and Integrin β1 in tumor tissues relative to the normal tissues (Fig. 6A,B). Quantitative analysis confirmed these findings, showing a marked increase in both proteins in CRC tissues (*p* < 0.001; Fig. 6C,D). A positive correlation between RAD23B and Integrin β1 expression was noted (*R* = 0.415; *p* < 0.0001; Fig. 6E), suggesting that RAD23B may regulate Integrin β1 expression. Based on immunohistochemical scores, Integrin β1 expression was classified as low (<0.6) or high (≥0.6), and all 90 patients were divided accordingly. Statistical analysis showed that high Integrin β1 expression was significantly related with lymph node metastasis (χ^2^ = 5.312, *p* = 0.021) and American Joint Committee on Cancer (AJCC) stage (χ^2^ = 4.416, *p* = 0.036), but not with sex age, tumor diameter, T stage, M stage, or vascular invasion (all *p* > 0.05) (Table 1). Univariate Cox proportional hazards regression analysis revealed that overall survival in CRC patients was significantly related with Integrin β1 expression, N stage, M stage, and AJCC stage. Multivariate Cox regression analysis identified N stage and Integrin β1 expression as independent prognostic factors for overall survival. Furthermore, elevated Integrin β1 expression was associated with a significantly poorer prognosis (*p* < 0.05) (Table 2). Kaplan-Meier survival analysis further confirmed that high Integrin β1 expression was linked to reduced overall survival in CRC patients (Fig. 6F).

**Table 2:** Univariate and multivariate Cox regression analysis of prognostic factors in overall survival of colorectal cancer patients

Clinicopathological parameters	Cox univariate analysis			Cox multivariate analysis		
	*HR*	95% *CI*	*p*	*HR*	95% *CI*	*p*
Sex (Male vs. Female)	0.945	0.521–1.713	0.851			
Age (≥60 vs. <60)	1.702	0.921–3.146	0.090			
Tumor diameter (≥7 cm vs. <7 cm)	0.853	0.412–1.765	0.668			
T stage (T3 + T4 vs. T1 + T2)	1.203	0.473–3.063	0.698			
N stage (N1-2 vs. N0)	3.021	1.646–5.547	<0.001	2.430	1.274–4.634	0.007
M stage (M1 vs. M0)	6.253	2.148–18.204	0.001	2.957	0.996–8.784	0.051
AJCC stage (III + IV vs. I + II)	3.185	1.712–5.928	<0.001			
Vascular invasion (Yes vs. No)	2.257	0.808–6.304	0.120			
Integrin β1 (High expression vs. Low expression)	3.899	2.091–7.271	<0.001	3.021	1.585–5.757	0.001

Note: HR, Hazard Ratio; CI, Confidence Interval; AJCC, American Joint Committee on Cancer.

## 4 Discussion

CRC is one of the most prevalent gastrointestinal malignancies worldwide, with metastasis remaining a critical factor influencing patient outcomes and overall survival [4]. In spite of recent improvements in diagnostic and therapeutic strategies for CRC, the high incidence of metastasis and the complex molecular mechanisms underlying disease progression contribute to persistently elevated mortality rates [23]. Existing literature indicates that aberrant gene expression and dysregulation are key drivers of cancer metastasis [24].

RAD23B, a critical gene involved in DNA damage repair, has emerged as a key player in driving tumor progression across several cancers, including breast and lung cancers [25,26]. Consistent with these findings, the present study confirmed that RAD23B is significantly upregulated in CRC tissues. Our previous research demonstrated that knockdown of RAD23B markedly suppressed CRC cell migration and invasion [18]. Furthermore, the current study revealed that AD23B upregulation led to a significant increase in CRC cell migration, invasion, and metastatic lesion formation, underscoring its pivotal role in promoting CRC metastasis.

Talin1, a cytoskeletal protein, plays a vital role in cell adhesion, integrin activation, and signal transduction. By binding to the intracellular domain of integrins, Talin1 directly influences tumor cell motility and invasiveness [13]. Previous studies have reported that elevated Talin1 expression is associated with increased tumor aggressiveness and advanced disease in several cancers, including skin cancer [27]. In this study, RAD23B overexpression notably upregulated Talin1 expression in CRC cells, and IP-MS analysis identified Talin1 and Integrin as RAD23B-interacting partners. These findings suggest that RAD23B may promote CRC metastasis by modulating Talin1/Integrin signaling. Given the well-established roles of Talin1 and Integrin in linking ECM remodeling to intracellular signaling pathways [28], our findings highlight a potential mechanism through which RAD23B facilitates CRC metastasis by enhancing cell-ECM interactions and downstream signaling cascades.

The PI3K/AKT signaling pathway is a critical intracellular cascade that regulates cell proliferation, survival, migration, and invasion. Sun et al. [29] reported that SLIT and NTRK-like family member 4 suppressed CRC proliferation and liver metastasis by modulating the PI3K/AKT/NFκB pathway and tumor-associated macrophages. Abnormal activation of the PI3K/AKT pathway has been observed in various malignancies and is strongly associated with increased tumor invasiveness and metastatic potential.

In the present study, transcriptomic analysis revealed that RAD23B overexpression notably upregulated the expression of PI3K, AKT, and MMP9, indicating that RAD23B may activate the PI3K/AKT pathway to drive CRC metastasis. Western blot analysis further confirmed elevated phosphorylation of PI3K and AKT, along with increased MMP9 expression in RAD23B-overexpressing cells, supporting the hypothesis that the PI3K/AKT/MMP9 axis plays a central role in RAD23B-mediated metastasis.

Inflammation is another critical factor influencing cancer metastasis. The TME in CRC is often characterized by chronic inflammation, where inflammatory mediators such as cytokines (e.g., TNF-α, IL-6), chemokines, and immune cells (e.g., macrophages) contribute to tumor progression and metastasis through activation of key signaling pathways, including PI3K/AKT [29,30]. These inflammatory signals may further enhance the activity of the PI3K/AKT pathway, which is already implicated in RAD23B-driven metastasis [31]. This interplay between inflammation and RAD23B signaling could amplify MMP9 expression, promoting ECM degradation and facilitating CRC cell invasion and metastatic colonization. Thus, chronic inflammation within the TME may exacerbate RAD23B’s pro-metastatic effects via the PI3K/AKT/MMP9 axis.

MMP9, a key member of the matrix metalloproteinase family, promotes tumor invasion and metastasis by degrading the ECM and basement membrane, thereby creating pathways for tumor cell migration and supporting angiogenesis [32]. Existing studies have reported that PI3K/AKT signaling can upregulate MMP9 expression, further enhancing tumor dissemination [33]. In line with these findings, the current study demonstrated that RAD23B overexpression notably increased MMP9 expression, suggesting that RAD23B promotes ECM degradation through MMP9 upregulation, enabling CRC cells to penetrate surrounding tissues and form distant metastases. For example, Xie et al. [34] reported that Wnt7A enhances EGF-induced migration in oral squamous cell carcinoma via activation of the PI3K/AKT/MMP9 pathway, while Thi et al. [35] showed that CD46 promotes bladder cancer metastasis by regulating MMP9 through activation of p38 MAPK, PI3K, and AKT pathways.

Immunohistochemical analysis revealed that both RAD23B and Integrin β1 proteins exhibited higher expression levels in tumor tissues than in surrounding healthy tissue. Their expression levels were positively correlated, and high Integrin β1 expression was notably related with lymph node metastasis, suggesting that RAD23B may enhance tumor metastatic potential by regulating Integrin β1. Furthermore, elevated Integrin β1 expression was closely associated with advanced clinical stage and poor prognosis. Therefore, combined detection of RAD23B and Integrin β1 expression may provide a more comprehensive assessment of metastatic risk and serve as a prognostic indicator in CRC patients.

Together with the IP-MS, transcriptomic, and western blotting results, these findings provide compelling evidence that RAD23B promotes CRC metastasis via the Talin1/Integrin αv/β1/PI3K/AKT/MMP9 signaling axis.

Importantly, the CTD serves as a valuable resource for identifying potential inhibitors of RAD23B by integrating chemical-gene-disease interaction data. In this study, L-Glutamine 21 was identified via GeneCards, which incorporates CTD data, as a compound inversely associated with RAD23B expression. Prior studies have demonstrated that glutamine can suppress tumor cell migration and invasion under specific conditions, supporting its potential anti-metastatic effects. For instance, Guo et al. [36] identified glutamine as a critical metabolic checkpoint that regulates tumor-immune cell interactions. Their study demonstrated that glutamine availability enhances conventional dendritic cell type 1 (cDC1)-mediated activation of cytotoxic CD8^+^ T cells, inhibits tumor progression, and restores immunotherapy sensitivity, mainly through competitive glutamine uptake via SLC38A2 and downstream FLCN-TFEB signaling.

Similarly, Pillai et al. [37] provided preclinical evidence supporting the therapeutic targeting of glutamine metabolism in cancer. Their work demonstrated that the glutamine antagonist prodrug DRP-104 significantly suppressed the growth of KEAP1-mutant lung tumors. The antitumor effects were attributed to inhibition of glutamine-dependent nucleotide synthesis and enhancement of antitumor immunity. Specifically, DRP-104 reversed T cell exhaustion, reduced regulatory T cells (Tregs), and improved the effector function of both CD4^+^ and CD8^+^ T cells, ultimately sensitizing tumors to anti-PD1 immune checkpoint blockade. These studies shed light the therapeutic potential of targeting glutamine metabolism to regulate tumor growth and immune responses, indirectly supporting the hypothesis that modulating glutamine pathways may inhibit CRC metastasis by downregulating RAD23B.

Although further experimental validation is needed, such toxicogenomic data mining provides a promising strategy for identifying novel inhibitors and advancing the understanding of RAD23B as a potential therapeutic target in metastatic CRC.

Nevertheless, it is critical to note the study limitations. CRC metastasis is influenced by multiple factors and complex regulatory networks. This study primarily focused on a single molecular pathway, and broader mechanistic insights remain unexplored. In addition, the clinical samples analyzed were obtained from a single medical center, with relatively homogeneous patient characteristics. Therefore, future studies involving multi-center cohorts and comprehensive pathway analyses are essential to validate and extend these findings.

## 5 Conclusions

In summary, the findings of this study demonstrate that RAD23B promotes CRC metastasis via the Talin1/Integrin αv/β1/PI3K/AKT/MMP9 signaling axis. As an upstream regulator, RAD23B facilitates ECM degradation by modulating key ECM-related factors, Talin1, Integrin β1, PI3K, AKT, and MMP9, thereby altering the TME and enhancing CRC cell invasion and metastasis. RAD23B overexpression was shown to upregulate Talin1 and Integrin expression, subsequently activating the PI3K/AKT pathway and leading to increased MMP9 expression. Elevated MMP9 levels promote ECM breakdown, enabling tumor cells to penetrate adjacent tissues and form distant metastatic lesions. These results provide innovative insights that illuminate the mechanisms behind CRC dissemination. RAD23B may serve not only as a prognostic biomarker but also as a promising therapeutic target. The development of RAD23B-targeted inhibitors or related therapeutic strategies holds potential for improving outcomes in patients with metastatic CRC.

## 6 Highlights

1. RAD23B promotes metastasis in CRC via a defined molecular mechanism.
2. Interactions within the Talin1/Integrin/PI3K/AKT/MMP9 axis were confirmed.
3. RAD23B and Integrin β1 expression predict CRC metastatic risk and prognosis.
4. Reference provided for RAD23B inhibitors and related targeted therapies.

## Supplementary Materials



## Data Availability

The data that support the findings of this study are available from the corresponding author upon reasonable request.

## Supplementary Materials

The supplementary material is available online at https://www.techscience.com/doi/10.32604/or.2025.067535/s1.

## Abbreviations

AKT: Protein kinase B
AJCC: American Joint Committee on Cancer
CCK-8: Cell Counting Kit-8
cDC1: Conventional dendritic cell type 1
CRC: Colorectal cancer
CTD: Comparative Toxicogenomics Database
DMP: Dimethyl pimelimidate
ECM: Extracellular matrix
FBS: Fetal bovine serum
GO: Gene Ontology
GSEA: Gene Set Enrichment Analysis
H&E: Hematoxylin and eosin
HRP: Horseradish peroxidase
IHC: Immunohistochemistry
IL-6: Interleukin 6
IP-MS: Immunoprecipitation-mass spectrometry
KEGG: Kyoto Encyclopedia of Genes and Genomes
MICs: Metastasis-initiating cells
MMP: Matrix metalloproteinase
MMP9: Matrix metalloproteinase 9
MOI: Multiplicity of infection
NCG: NOD/ShiLtJGpt-Prkdc^em26Cd52^Il2rg^em26Cd22^/Gpt (mouse strain)
OE: Overexpression
PBS: Phosphate-buffered saline
PBST: PBS + Tween 20
PI3K: Phosphoinositide 3-kinase
PVDF: Polyvinylidene fluoride
RAD23B: Radiation sensitive 23 homolog B
RNA-seq: RNA sequencing
RT: Room temperature
SPF: Specific pathogen-free
SRA: Sequence Read Archive
TACE: Transarterial chemoembolization
TME: Tumor microenvironment
TNF-α: Tumor necrosis factor alpha
Tregs: Regulatory T cells
Tris: Tris(hydroxymethyl)aminomethane
WB: Western blot
