# Supplementary material for: RAD23B Promotes Colorectal Cancer Metastasis via the Talin1/Integrin/PI3K/AKT/MMP9 Axis
Source: Oncol Res. 2025 Oct 22;33(11):3523–41. doi: 10.32604/or.2025.067535 (PMC12573188; doi:10.32604/or.2025.067535)
Supplement: Supplementary file 1 [file OncolRes-33-67535-s001.docx]

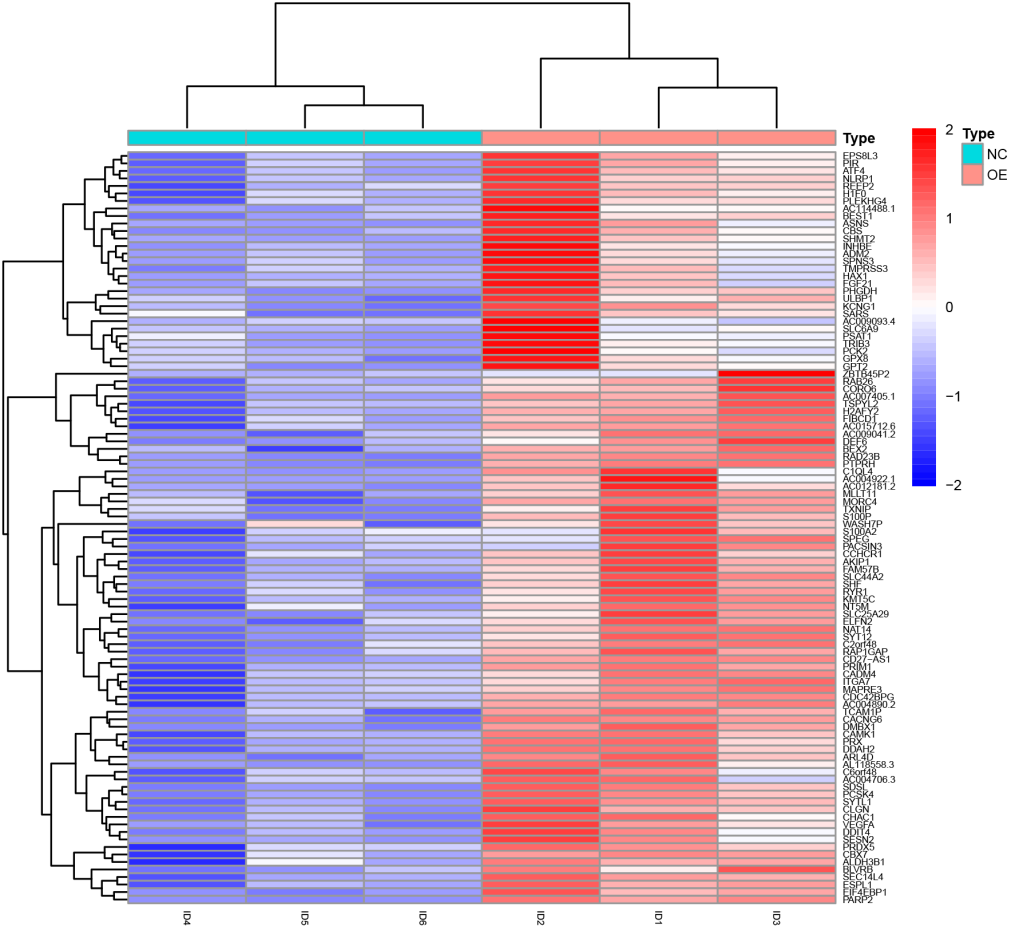


**Figure S1:** The top 100 differentially expressed genes (UP) heat map of the transcriptome analysis.


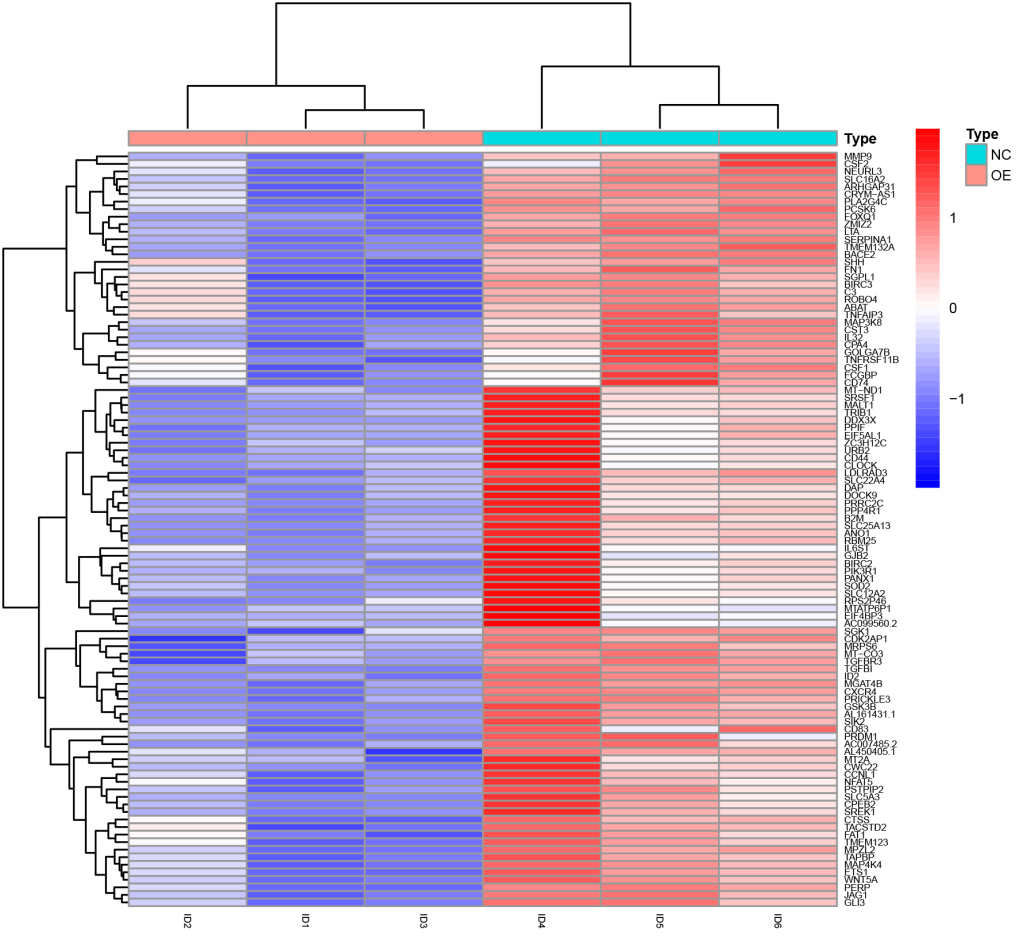


**Figure S2:** The top 100 differentially expressed genes (Down) heat map of the transcriptome analysis.


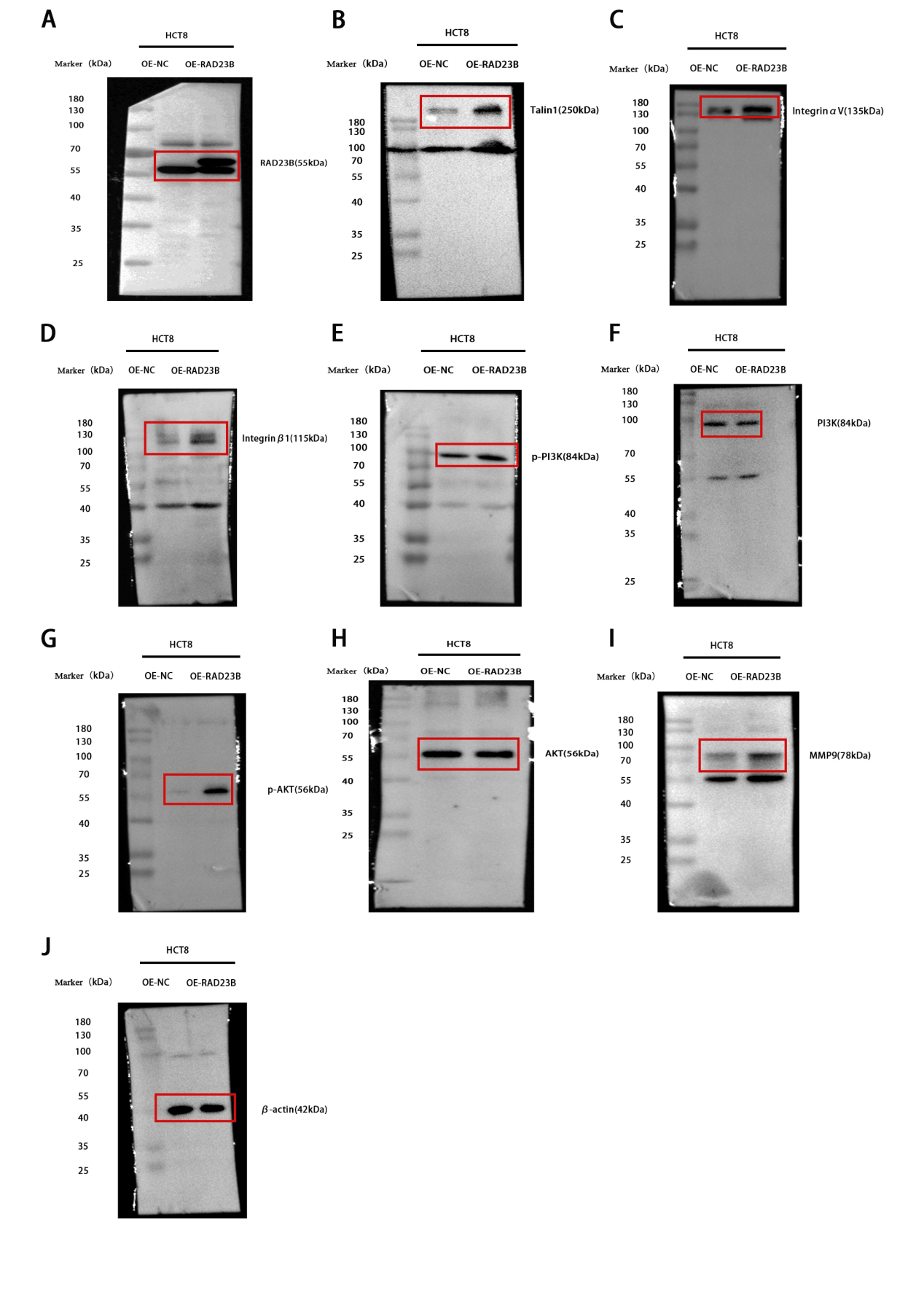


**Figure S3:** RAD23B promotes colorectal cancer progression via the Talin1/Integrin/PI3K/AKT/MMP9 signaling axis in HCT8 cells. (**A**–**J**) Western blot analysis of signaling molecules in HCT8 colorectal cancer cells transfected with RAD23B overexpression plasmid (OE-RAD23B) or negative control plasmid (OE-NC). (**A**) RAD23B: Overexpression of RAD23B.(**B**) Talin1: Expression was elevated in the OE-RAD23B group, indicating potential regulation by RAD23B. (**C**) Integrin αv: Upregulated upon RAD23B overexpression, suggesting enhanced cell-extracellular matrix (ECM) interactions. (**D**) Integrin β1: Increased expression in RAD23B-overexpressing cells, correlated with metastatic potential. (**E**) PI3K: Total PI3K protein levels showed no significant change between groups. (**F**) p-PI3K: Phosphorylated PI3K was markedly upregulated in the OE-RAD23B group, indicating pathway activation. (**G**) AKT: Total AKT protein levels showed no significant change between groups. (**H**) p-AKT: Phosphorylation of AKT was significantly increased in the OE-RAD23B group. (**I**) MMP9: Elevated expression suggests enhanced ECM degradation and pro-metastatic capacity. (**J**) β-actin: Internal reference.


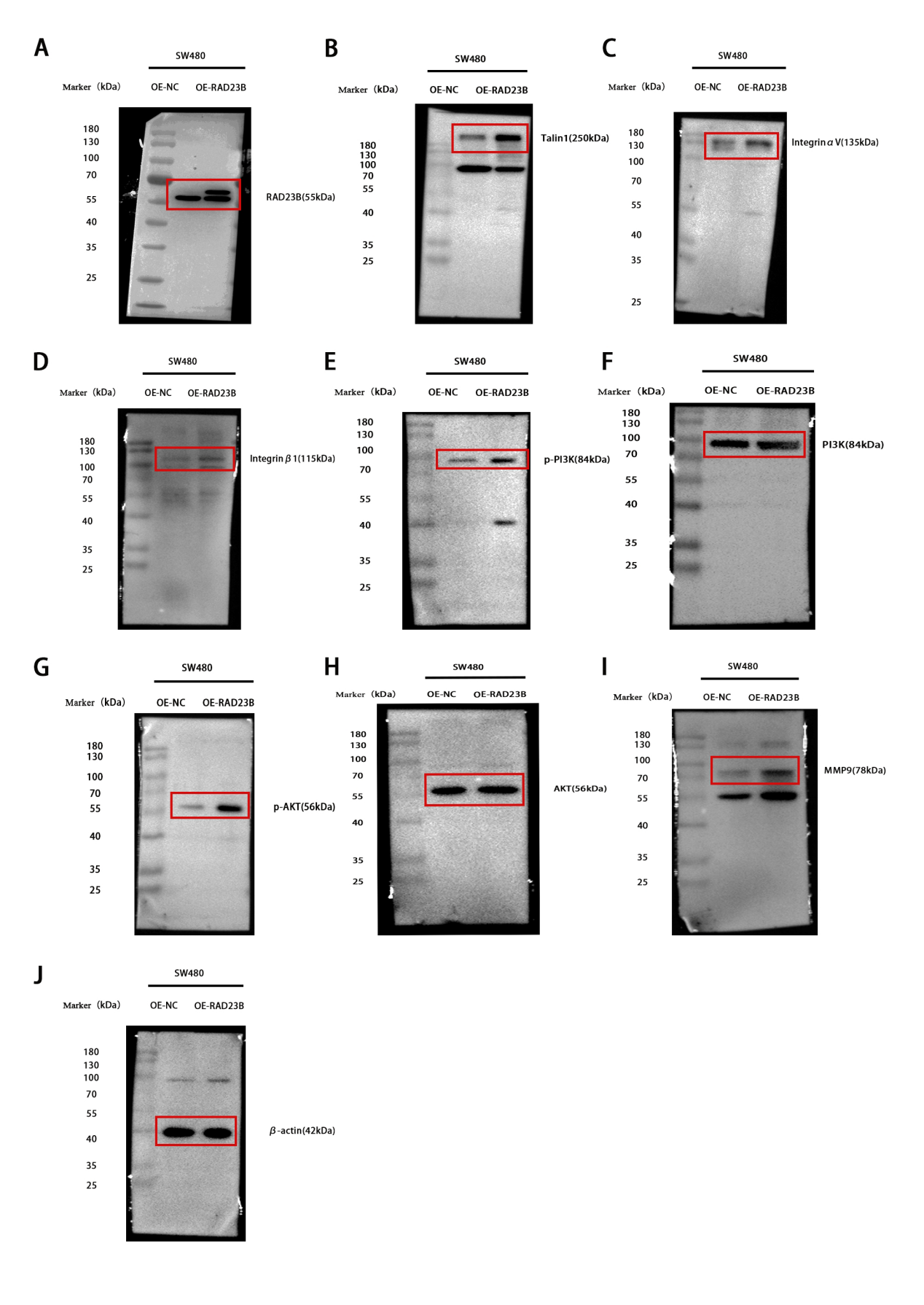


**Figure S4:** RAD23B promotes colorectal cancer progression via the Talin1/Integrin/PI3K/AKT/MMP9 signaling axis in SW480 cells. (**A**–**J**) Western blot analysis of signaling molecules in SW480 colorectal cancer cells transfected with RAD23B overexpression plasmid (OE-RAD23B) or negative control plasmid (OE-NC). (**A**) RAD23B: Overexpression of RAD23B. (**B**) Talin1: Expression was elevated in the OE-RAD23B group, indicating potential regulation by RAD23B. (**C**) Integrin αv: Upregulated upon RAD23B overexpression, suggesting enhanced cell–extracellular matrix (ECM) interactions. (**D**) Integrin β1: Increased expression in RAD23B-overexpressing cells, correlated with metastatic potential. (**E**) PI3K: Total PI3K protein levels showed no significant change between groups. (**F**) p-PI3K: Phosphorylated PI3K was markedly upregulated in the OE-RAD23B group, indicating pathway activation. (**G**) AKT: Total AKT protein levels showed no significant change between groups. (**H**) p-AKT: Phosphorylation of AKT was significantly increased in the OE-RAD23B group. (**I**) MMP9: Elevated expression suggests enhanced ECM degradation and pro-metastatic capacity. (**J**) β-actin: Internal reference.

**Supplementary Table S1:** The top interacting RAD23B interacting proteins identified by IP-MS.

| **Accession** | **Protein Names** | **Gene Names** | **Protein Score** | **#PSMs** |
| --- | --- | --- | --- | --- |
| P54727 | UV excision repair protein RAD23 homolog B | RAD23B | 802.72 | 18 |
| Q9Y2X3 | Nucleolar protein 58 | NOP58 | 573.70 | 13 |
| Q96P16 | Regulation of nuclear pre-mRNA domain-containing protein 1A | RPRD1A | 571.71 | 13 |
| P52597 | Heterogeneous nuclear ribonucleoprotein F | HNRNPF | 428.16 | 8 |
| Q9NQG5 | Regulation of nuclear pre-mRNA domain-containing protein 1B | RPRD1B | 392.11 | 9 |
| Q9BZE4 | Nucleolar GTP-binding protein 1 | GTPBP4 | 386.58 | 9 |
| Q01831 | DNA repair protein complementing XP-C cells | XPC | 381.18 | 10 |
| O76021 | Ribosomal L1 domain-containing protein 1 | RSL1D1 | 367.08 | 8 |
| O75367 | Core histone macro-H2A.1 | MACROH2A1 | 355.01 | 7 |
| Q9H0A0 | RNA cytidine acetyltransferase | NAT10 | 333.60 | 8 |
| Q92841 | Probable ATP-dependent RNA helicase DDX17 | DDX17 | 332.90 | 9 |
| P08708 | 40S ribosomal protein S17 | RPS17 | 326.87 | 5 |
| Q9NVP1 | ATP-dependent RNA helicase DDX18 | DDX18 | 315.50 | 8 |
| Q9BVP2 | Guanine nucleotide-binding protein-like 3 | GNL3 | 262.25 | 4 |
| P15531 | Nucleoside diphosphate kinase A | NME1 | 261.73 | 7 |
| Q9BUH6 | Protein PAXX | PAXX | 254.61 | 5 |
| Q15436 | Protein transport protein Sec23A | SEC23A | 252.82 | 4 |
| P05387 | 60S acidic ribosomal protein P2 | RPLP2 | 246.78 | 4 |
| Q6P5R6 | 60S ribosomal protein L22-like 1 | RPL22L1 | 240.49 | 6 |
| O43818 | U3 small nucleolar RNA-interacting protein 2 | RRP9 | 235.80 | 5 |
| P02533 | Keratin, type I cytoskeletal 14 | KRT14 | 225.61 | 7 |
| O00159 | Unconventional myosin-Ic | MYO1C | 224.97 | 5 |
| Q99733 | Nucleosome assembly protein 1-like 4 | NAP1L4 | 223.86 | 5 |
| Q9H0U4 | Ras-related protein Rab-1B | RAB1B | 213.18 | 5 |
| P61006 | Ras-related protein Rab-8A | RAB8A | 212.80 | 5 |
| Q01130 | Serine/arginine-rich splicing factor 2 | SRSF2 | 209.78 | 5 |
| Q8IUE6 | Histone H2A type 2-B | H2AC21 | 207.64 | 7 |
| P13804 | Electron transfer flavoprotein subunit alpha, mitochondrial | ETFA | 205.69 | 4 |
| Q13426 | DNA repair protein XRCC4 | XRCC4 | 203.76 | 3 |
| P21796 | Voltage-dependent anion-selective channel protein 1 | VDAC1 | 202.48 | 5 |
| P41208 | Centrin-2 | CETN2 | 202.09 | 4 |
| Q9Y5B9 | FACT complex subunit SPT16 | SUPT16H | 199.21 | 7 |
| Q9Y2W1 | Thyroid hormone receptor-associated protein 3 | THRAP3 | 199.11 | 7 |
| Q9NX58 | Cell growth-regulating nucleolar protein | LYAR | 194.23 | 5 |
| O00232 | 26S proteasome non-ATPase regulatory subunit 12 | PSMD12 | 192.05 | 5 |
| Q15397 | Pumilio homolog 3 | PUM3 | 191.43 | 5 |
| P16403 | Histone H1.2 | H1-2 | 184.99 | 5 |
| O76094 | Signal recognition particle subunit SRP72 | SRP72 | 184.42 | 4 |
| O75475 | PC4 and SFRS1-interacting protein | PSIP1 | 181.66 | 4 |
| Q8TDN6 | Ribosome biogenesis protein BRX1 homolog | BRIX1 | 180.74 | 4 |
| Q15050 | Ribosome biogenesis regulatory protein homolog | RRS1 | 180.30 | 4 |
| Q06830 | Peroxiredoxin-1 | PRDX1 | 175.85 | 6 |
| O60832 | H/ACA ribonucleoprotein complex subunit DKC1 | DKC1 | 175.30 | 6 |
| O00541 | Pescadillo homolog | PES1 | 171.69 | 4 |
| P25398 | 40S ribosomal protein S12 | RPS12 | 171.39 | 4 |
| P62136 | Serine/threonine-protein phosphatase PP1-alpha catalytic subunit | PPP1CA | 170.69 | 4 |
| O00425 | Insulin-like growth factor 2 mRNA-binding protein 3 | IGF2BP3 | 164.12 | 3 |
| Q1KMD3 | Heterogeneous nuclear ribonucleoprotein U-like protein 2 | HNRNPUL2 | 162.17 | 3 |
| Q15269 | Periodic tryptophan protein 2 homolog | PWP2 | 161.61 | 4 |
| Q9NXF1 | Testis-expressed protein 10 | TEX10 | 161.53 | 3 |
| Q96QR8 | Transcriptional activator protein Pur-beta | PURB | 161.45 | 3 |
| P38919 | Eukaryotic initiation factor 4A-III | EIF4A3 | 161.37 | 5 |
| P04908 | Histone H2A type 1-B/E | H2AC4 | 160.47 | 4 |
| P62913 | 60S ribosomal protein L11 | RPL11 | 159.90 | 4 |
| P41091 | Eukaryotic translation initiation factor 2 subunit 3 | EIF2S3 | 158.08 | 5 |
| Q9Y262 | Eukaryotic translation initiation factor 3 subunit L | EIF3L | 151.92 | 4 |
| P26368 | Splicing factor U2AF 65 kDa subunit | U2AF2 | 149.09 | 4 |
| P62995 | Transformer-2 protein homolog beta | TRA2B | 148.01 | 3 |
| Q5SSJ5 | Heterochromatin protein 1-binding protein 3 | HP1BP3 | 147.51 | 3 |
| O60264 | SWI/SNF-related matrix-associated actin-dependent regulator of chromatin subfamily A member 5 | SMARCA5 | 146.81 | 5 |
| O94925 | Glutaminase kidney isoform, mitochondrial | GLS | 144.73 | 4 |
| Q14137 | Ribosome biogenesis protein BOP1 | BOP1 | 143.79 | 3 |
| P20042 | Eukaryotic translation initiation factor 2 subunit 2 | EIF2S2 | 143.01 | 3 |
| Q92522 | Histone H1.10 | H1-10 | 140.04 | 3 |
| P62266 | 40S ribosomal protein S23 | RPS23 | 139.81 | 5 |
| O75533 | Splicing factor 3B subunit 1 | SF3B1 | 138.95 | 4 |
| Q16643 | Drebrin | DBN1 | 136.66 | 3 |
| Q07020 | 60S ribosomal protein L18 | RPL18 | 134.43 | 4 |
| Q96SB4 | SRSF protein kinase 1 | SRPK1 | 132.59 | 4 |
| P16401 | Histone H1.5 | H1-5 | 130.52 | 4 |
| Q15555 | Microtubule-associated protein RP/EB family member 2 | MAPRE2 | 130.51 | 4 |
| P55884 | Eukaryotic translation initiation factor 3 subunit B | EIF3B | 128.56 | 3 |
| Q15393 | Splicing factor 3B subunit 3 | SF3B3 | 126.68 | 3 |
| P62851 | 40S ribosomal protein S25 | RPS25 | 125.97 | 3 |
| P33993 | DNA replication licensing factor MCM7 | MCM7 | 122.51 | 3 |
| Q8IY81 | pre-rRNA 2'-O-ribose RNA methyltransferase FTSJ3 | FTSJ3 | 122.22 | 4 |
| P29373 | Cellular retinoic acid-binding protein 2 | CRABP2 | 119.31 | 3 |
| P51571 | Translocon-associated protein subunit delta | SSR4 | 119.22 | 3 |
| Q96GQ7 | Probable ATP-dependent RNA helicase DDX27 | DDX27 | 118.43 | 4 |
| O43795 | Unconventional myosin-Ib | MYO1B | 117.60 | 3 |
| Q9ULV4 | Coronin-1C | CORO1C | 115.19 | 3 |
| P61088 | Ubiquitin-conjugating enzyme E2 N | UBE2N | 112.80 | 4 |
| Q13435 | Splicing factor 3B subunit 2 | SF3B2 | 112.13 | 4 |
| Q07666 | KH domain-containing, RNA-binding, signal transduction-associated protein 1 | KHDRBS1 | 109.63 | 4 |
| O00764 | Pyridoxal kinase | PDXK | 107.75 | 3 |
| Q9P035 | Very-long-chain (3R)-3-hydroxyacyl-CoA dehydratase 3 | HACD3 | 107.37 | 3 |
| P53618 | Coatomer subunit beta | COPB1 | 106.08 | 3 |
| Q9UHB6 | LIM domain and actin-binding protein 1 | LIMA1 | 102.72 | 3 |
| Q9NVI7 | ATPase family AAA domain-containing protein 3A | ATAD3A | 98.80 | 3 |
| P46087 | Probable 28S rRNA (cytosine(4447)-C(5))-methyltransferase | NOP2 | 97.81 | 3 |
| Q9Y3U8 | 60S ribosomal protein L36 | RPL36 | 95.30 | 3 |
| Q8WTT2 | Nucleolar complex protein 3 homolog | NOC3L | 94.65 | 3 |
| P50416 | Carnitine O-palmitoyltransferase 1, liver isoform | CPT1A | 94.45 | 3 |
| O60841 | Eukaryotic translation initiation factor 5B | EIF5B | 93.88 | 3 |
| P18621 | 60S ribosomal protein L17 | RPL17 | 93.77 | 3 |
| Q14978 | Nucleolar and coiled-body phosphoprotein 1 | NOLC1 | 91.94 | 3 |
| Q96HS1 | Serine/threonine-protein phosphatase PGAM5, mitochondrial | PGAM5 | 86.87 | 3 |
| P30876 | DNA-directed RNA polymerase II subunit RPB2 | POLR2B | 86.57 | 3 |
| O60884 | DnaJ homolog subfamily A member 2 | DNAJA2 | 85.93 | 3 |
| P62873 | Guanine nucleotide-binding protein G(I)/G(S)/G(T) subunit beta-1 | GNB1 | 85.75 | 3 |
| P12236 | ADP/ATP translocase 3 | SLC25A6 | 85.41 | 3 |
| P53992 | Protein transport protein Sec24C | SEC24C | 80.85 | 3 |
| P62280 | 40S ribosomal protein S11 | RPS11 | 78.92 | 3 |
| Q02543 | 60S ribosomal protein L18a | RPL18A | 74.43 | 3 |
| P62829 | 60S ribosomal protein L23 | RPL23 | 73.27 | 3 |

**Supplementary Table S2:** The top100 differentially expressed genes (UP).

| **Gene Name** | **OE_1** | **OE_2** | **OE_3** | **OENC_1** | **OENC_2** | **OENC_3** | **log2FoldChange** | ***p*-value** |
| --- | --- | --- | --- | --- | --- | --- | --- | --- |
| RAD23B | 12563.0577023506 | 11976.937909583 | 13250.6153023234 | 7129.83118676997 | 6625.37728474732 | 6547.5337713384 | 0.89636818369748 | 5.88772856151094e-19 |
| CBS | 1178.77255513266 | 1622.5795313082 | 936.271021361776 | 534.247440293193 | 566.474401078691 | 683.220915270094 | 1.06678224743971 | 8.45964186813684e-09 |
| DDIT4 | 959.843994609238 | 1128.66473510314 | 654.59626493514 | 346.538880190179 | 495.278164877544 | 416.215040336954 | 1.12469410776629 | 9.4183626163709e-09 |
| CHAC1 | 368.067667255534 | 376.882776220731 | 242.994068044105 | 120.669788637652 | 187.792970849402 | 117.796709529327 | 1.21358065798395 | 1.79301967517464e-07 |
| SESN2 | 717.01493621208 | 837.076481921834 | 537.562387264918 | 366.134828772362 | 399.31802043252 | 375.967831247767 | 0.873883494151709 | 3.18227719239098e-07 |
| KCNG1 | 331.738910487456 | 383.825353677429 | 275.723881290862 | 196.990851536679 | 157.869915054717 | 146.26424766558 | 0.984755214682346 | 4.57171921974548e-07 |
| PHGDH | 4156.77459020017 | 5382.48112249976 | 4193.38334567541 | 3168.3554760245 | 2953.09605808236 | 2970.44035863118 | 0.594939756468753 | 2.82915364478163e-06 |
| SYTL1 | 887.18648107308 | 966.010063260505 | 833.12251900836 | 522.902417429824 | 631.479660218869 | 585.056990662322 | 0.62696920271417 | 3.22528647141039e-06 |
| INHBE | 139.578907582618 | 278.694895047435 | 122.984752805996 | 54.6623828871414 | 78.4190427722779 | 66.751468733285 | 1.43718324787602 | 3.3235357956393e-06 |
| ADM2 | 586.040207864007 | 1024.52607325267 | 475.078198339291 | 312.503811600072 | 380.745089249612 | 295.473413069394 | 1.07694537955622 | 3.59388981234181e-06 |
| TXNIP | 7548.73324844078 | 5447.93970994862 | 6489.42933556155 | 4914.45763126846 | 3562.90729858784 | 3774.40290116884 | 0.669507487719792 | 1.05973557817378e-05 |
| CACNG6 | 327.914830827658 | 334.235514701017 | 319.363632286538 | 186.677194388162 | 219.77968566441 | 185.529817508689 | 0.729802649744681 | 1.1489892910008e-05 |
| CD27-AS1 | 299.234233379175 | 278.694895047435 | 295.560131743442 | 159.861685802017 | 180.570164278271 | 193.382931477311 | 0.710015591643074 | 3.94139447145227e-05 |
| ASNS | 499.998415518557 | 614.914003307509 | 391.765946438455 | 318.692005889183 | 302.326046477334 | 295.473413069394 | 0.717279729498223 | 5.91369583900477e-05 |
| C6orf48 | 1312.61534322558 | 1501.58032420576 | 912.46752081868 | 465.145937398127 | 838.877391761341 | 782.366479123944 | 0.836770682825867 | 7.94679904607374e-05 |
| SHF | 314.530552018366 | 237.039430307249 | 260.846693451427 | 143.35983436439 | 188.824800359564 | 142.33769068127 | 0.77663622265641 | 9.74276794686234e-05 |
| DMBX1 | 79.3496529408035 | 68.4339777874485 | 66.451439016143 | 29.90960573 | 31.9867148150081 | 27.4858988901762 | 1.26227857617683 | 9.92175958601925e-05 |
| ULBP1 | 158.699305881607 | 250.924585220645 | 188.44437929951 | 128.920714356465 | 100.08746248567 | 79.5127789322954 | 0.955653458305731 | 0.000112984403288768 |
| SHMT2 | 9207.42780087806 | 11638.7352077639 | 8501.81694397579 | 7034.94554100361 | 6983.42212477338 | 6905.83209615677 | 0.488082765453024 | 0.000141782824616059 |
| PRX | 430.208961727248 | 419.530037740445 | 356.060695623811 | 221.743628693121 | 272.402990682649 | 281.730463624306 | 0.635851732405282 | 0.000145053917313782 |
| CAMK1 | 144.359007157365 | 141.826939472538 | 120.009315238109 | 42.2859943089207 | 79.4508722824394 | 76.5678611940622 | 1.03364148814086 | 0.000154579929607982 |
| NAT14 | 929.251357330856 | 818.232343110798 | 919.410208477083 | 579.627531746669 | 630.447830708708 | 687.147472254405 | 0.491085798679242 | 0.000193758961770317 |
| ARL4D | 530.591052796939 | 497.881983323176 | 460.201010499856 | 346.538880190179 | 322.962636680565 | 360.261603310524 | 0.531655621367164 | 0.000244778757438435 |
| RYR1 | 356.595428276141 | 261.834349795455 | 309.445507060248 | 157.798954372314 | 224.938833215218 | 181.603260524378 | 0.717843965896666 | 0.000254464800217868 |
| SDSL | 138.622887667669 | 145.794126590651 | 118.025690192851 | 71.1642343247689 | 78.4190427722779 | 73.6229434558291 | 0.850605282653065 | 0.0002990600407086 |
| DDAH2 | 1102.2909619367 | 1097.91903493776 | 972.968084699049 | 680.701371802138 | 804.82701792601 | 805.925821029809 | 0.469584136450494 | 0.000310649739264211 |
| PRDX5 | 2407.25814584269 | 2556.8520976238 | 2151.2413615823 | 1166.4746234973 | 1830.46555102659 | 1787.56506710753 | 0.572477649238334 | 0.000439385987371132 |
| EIF4EBP1 | 1731.35206597344 | 1924.08575228479 | 1770.38535289277 | 1393.37508076468 | 1347.56934027099 | 1410.61559661369 | 0.386102735733559 | 0.00044492049339003 |
| SLC6A9 | 531.547072711889 | 1103.86981561493 | 600.046576190545 | 472.365497402089 | 435.432053288175 | 397.563894661477 | 0.776068402495254 | 0.000544424087773649 |
| PTPRH | 161.567365626455 | 144.802329811123 | 162.657253711156 | 97.9797429109138 | 92.8646559145396 | 90.3108106391503 | 0.738686497475598 | 0.000566480811722442 |
| RAB26 | 237.092938907461 | 206.293730141874 | 284.650193994523 | 118.607057207948 | 169.220039666494 | 156.080640126358 | 0.713434654034918 | 0.000659759843696774 |
| KMT5C | 833.649365835912 | 661.528451945336 | 775.597392695878 | 470.302765972386 | 576.792696180307 | 561.497648756457 | 0.497405350000855 | 0.000732830132867699 |
| ATF4 | 9046.81645516655 | 10635.0368668813 | 8638.68707209859 | 6661.59115222728 | 7674.74789658162 | 7168.9114141056 | 0.397152354583191 | 0.000746039165660144 |
| NLRP1 | 217.972540608472 | 289.604659622246 | 214.231504887864 | 118.607057207948 | 169.220039666494 | 153.135722388124 | 0.710544401831559 | 0.00077667303932203 |
| FIBCD1 | 881.450361583384 | 824.183123787967 | 929.328333703373 | 554.874754590227 | 714.026021031793 | 650.826820149529 | 0.456842830196903 | 0.000964348914864541 |
| H1F0 | 8486.58878500618 | 10106.4091833928 | 7812.50724074864 | 5340.41167150223 | 7184.62887925488 | 6680.05506955889 | 0.459333284192944 | 0.00102070985901813 |
| ITGA7 | 2776.28183301317 | 2477.50835526154 | 2831.6247521058 | 1755.38444667763 | 2155.49184672748 | 2173.34929081607 | 0.410191146372837 | 0.00102166370884695 |
| AKIP1 | 619.500904887237 | 528.627683488552 | 541.529637355434 | 358.9152688 | 414.795463084943 | 434.86618601243 | 0.483329014913278 | 0.00106446164345334 |
| C1QL4 | 48.7570156624214 | 37.6882776220731 | 23.803500543096 | 12.3763885782207 | 11.3501246117771 | 12.7613101990104 | 1.59585012 | 0.00108514949933974 |
| ZBTB45P2 | 3.82407965979776 | 3.96718711811296 | 27.770750633612 | 1.18163924606653 | 0.8815633456771442 | 0.981639246077721 | 5.02586991616793 | 0.00111245731818951 |
| SYT12 | 270.553635930691 | 216.211697937156 | 263.822131019314 | 147.485297223796 | 165.092721625848 | 178.658342786145 | 0.611332211609337 | 0.00115471489137076 |
| SEC14L4 | 572.655929054715 | 616.897596866565 | 563.349512853272 | 386.762143069396 | 441.623030349144 | 453.517331687907 | 0.451178378894253 | 0.00122044307957826 |
| C2orf48 | 125.238608858377 | 109.097645748106 | 127.943815419141 | 53.6310171722896 | 61.9097706096931 | 86.3842536548395 | 0.841497444076579 | 0.00135988424088208 |
| VEGFA | 1019.1172293361 | 1141.558093 | 934.287396316518 | 776.618383283348 | 816.177142537787 | 728.376320589669 | 0.415200040093254 | 0.00139325506178659 |
| TRIB3 | 2195.97774463886 | 3312.60124362432 | 2096.69167283771 | 1931.74798391728 | 1688.0730786243 | 1642.28245868803 | 0.531357189867084 | 0.00139791468050782 |
| SLC44A2 | 653.917621825417 | 558.381586874399 | 612.940138984722 | 425.954040233762 | 473.609745164152 | 444.682578473208 | 0.441476330219553 | 0.00140391877073696 |
| TSPYL2 | 926.383297586007 | 893.608898354944 | 1029.5013984889 | 606.443040332814 | 756.331030948417 | 740.155991542602 | 0.43813416523078 | 0.00140508002341251 |
| MLLT11 | 243.785078312107 | 200.342949464704 | 218.19875497838 | 156.767588657462 | 120.724052688901 | 151.172443895969 | 0.627660403099617 | 0.00150502695267938 |
| CBX7 | 884.318421328232 | 869.805775646266 | 862.87689468723 | 521.871051714972 | 723.312486623247 | 657.698294872073 | 0.459659185178962 | 0.0015104984568983 |
| NT5M | 318.354631678164 | 260.842553015927 | 294.568319220813 | 142.328468649538 | 229.066151255864 | 190.438013739078 | 0.637237943768306 | 0.00155649625780497 |
| AC009093.4 | 54.4931351521181 | 193.400372008007 | 36.697063337273 | 22.6900457267379 | 39.2095213861389 | 35.339012858798 | 1.54845462173968 | 0.0015743694144655 |
| SLC25A29 | 2993.2983537067 | 2464.61499712768 | 2727.48443722975 | 2014.25724110542 | 2067.78633836375 | 2245.99059502583 | 0.371283366488645 | 0.00159244765431514 |
| SPEG | 496.174335858759 | 341.178092157715 | 473.094573294033 | 226.900457267379 | 307.485194028142 | 327.867508189959 | 0.60365641734734 | 0.00182385800739569 |
| AC015712.6 | 492.350256198962 | 490.939405866479 | 518.717949334967 | 305.28425159611 | 402.413508963005 | 371.059635017379 | 0.477368332065151 | 0.00182716921815869 |
| PSAT1 | 4389.08742953288 | 7511.86880814689 | 4361.99147452234 | 4263.66586519703 | 3349.31858998439 | 3170.69476483104 | 0.592745709968264 | 0.0018273177766795 |
| AC009041.2 | 67.8774139614102 | 49.589838976412 | 69.42687658 | 28.8782400158483 | 20.636590203231 | 36.3206521048757 | 1.12066207838608 | 0.00185303536804942 |
| AC007405.1 | 28.6805974484832 | 30.7457001653754 | 37.688875859902 | 7.21956000396207 | 13.4137836321002 | 12.7613101990104 | 1.53818964398569 | 0.00209467041608267 |
| MAPRE3 | 372.847766830282 | 332.25192114196 | 384.823258780052 | 190.802657247569 | 267.243843131842 | 280.748824378228 | 0.560483482886566 | 0.00210969240865371 |
| MORC4 | 722.751055701777 | 678.388997197316 | 683.358828091381 | 576.533434602114 | 472.57791565399 | 512.41568645257 | 0.416869383186155 | 0.00214268762246619 |
| BLVRB | 928.295337415906 | 1060.23075731569 | 1103.88733768608 | 737.426486118982 | 773.872132621163 | 840.283194642529 | 0.394779011860001 | 0.00218078166122006 |
| PLEKHG4 | 96.5580114098934 | 150.753110488292 | 96.205814695013 | 34.0350685901069 | 76.3553837519548 | 63.8065509950519 | 0.978815714587135 | 0.00224219585095316 |
| HAX1 | 1161.56419666357 | 1524.3916501349 | 949.164584155953 | 839.531691889303 | 864.67312951538 | 870.714011270939 | 0.497424227946279 | 0.00224406292420268 |
| ESPL1 | 2847.98332663438 | 3078.53720365566 | 2914.93700400663 | 2189.58941263021 | 2456.78606369465 | 2405.99779213649 | 0.326123260740064 | 0.00234876611909309 |
| CDC42BPG | 869.022102689041 | 812.281562433628 | 851.966956938311 | 562.094314594189 | 676.880158665977 | 682.239276024016 | 0.398842712485781 | 0.00264093458196397 |
| PCSK4 | 173.039604605849 | 185.465997771781 | 156.706378575382 | 106.230668629728 | 118.660393668578 | 110.925234806782 | 0.617584393419505 | 0.00267580424354644 |
| REEP2 | 441.681200706641 | 537.553854504306 | 429.454822298357 | 267.123720146596 | 345.66288590412 | 372.041274263456 | 0.515862743400378 | 0.00273526303659504 |
| CCHCR1 | 1199.80499326155 | 1014.60810545739 | 995.779772719516 | 666.262251794214 | 895.628014820226 | 808.870738768042 | 0.437394541435734 | 0.00275800672741354 |
| AC004922.1 | 10.5162190644438 | 4.9589838976412 | 2.975437568 | 0.9815633745275232 | 1.081639246077721 | 0.9515445625897 | 5.0440626610723 | 0.00279362787416027 |
| EPS8L3 | 770.552051449249 | 889.641711236831 | 700.219640976074 | 537.341537437748 | 625.2886832 | 599.781579353488 | 0.421400546465668 | 0.00298882323524132 |
| AC012181.2 | 25.8125377036349 | 14.8769516929236 | 13.885375316806 | 5.15682857425862 | 4.1273180406462 | 2.94491773823316 | 2.16273671679268 | 0.00302172783471258 |
| RAP1GAP | 173.995624520798 | 145.794126590651 | 151.747315962237 | 83.5406229029896 | 94.9283149348627 | 117.796709529327 | 0.669610811848084 | 0.00309190146319307 |
| AC004890.2 | 150.095126647062 | 145.794126590651 | 158.69000362064 | 68.0701371802138 | 103.182951016155 | 107.980317068549 | 0.70207555035766 | 0.00311856633479564 |
| PARP2 | 595.600407013501 | 629.790955000432 | 616.907389075238 | 473.396863116941 | 466.386938593021 | 474.131755855539 | 0.381713796787694 | 0.00312473625274094 |
| TCAM1P | 86.997812260399 | 78.3519455827309 | 73.394126674546 | 41.2546285940689 | 53.6551345284007 | 33.3757343666425 | 0.898135249874022 | 0.00336502346235671 |
| DEF6 | 420.648762577754 | 357.046840630166 | 464.168260590372 | 283.625571584224 | 292.007751375719 | 320.014394221337 | 0.471240271507125 | 0.00350940116329887 |
| TMPRSS3 | 152.96318639191 | 217.203494716685 | 111.083002534448 | 74.2583314693241 | 106.27843954664 | 97.1822853616944 | 0.79283598067009 | 0.00354489744279329 |
| S100A2 | 3008.59467234589 | 1959.7904363478 | 2388.28455449063 | 1174.72554921611 | 1830.46555102659 | 1998.61750501424 | 0.555979407191846 | 0.00354990782718525 |
| GPX8 | 529.63503288199 | 723.019852276087 | 489.955386178726 | 445.549988815945 | 423.050099166236 | 354.371767834057 | 0.511079112734648 | 0.00363126922011812 |
| BEX2 | 205.54428171413 | 199.351152685176 | 221.174192546267 | 153.673491512907 | 114.533075627932 | 151.172443895969 | 0.577546744331937 | 0.00378729348606395 |
| SPNS3 | 112.810349964034 | 180.50701387414 | 89.26312704 | 66.0074057505103 | 82.5463608129241 | 64.7881902411296 | 0.842798422235103 | 0.00395771650009535 |
| PIR | 286.805974484832 | 333.243717921489 | 252.912193270395 | 173.26944009509 | 224.938833215218 | 204.180963184166 | 0.535114297115539 | 0.00400590664860936 |
| ELFN2 | 934.987476820552 | 807.322578535987 | 867.835957300375 | 658.0113261 | 619.097706096931 | 732.30287757398 | 0.377212934054578 | 0.00426727414982025 |
| GPT2 | 1643.39823379809 | 2120.46151463138 | 1558.13747305016 | 1454.22565794093 | 1271.21395651903 | 1300.67200105298 | 0.402567772811161 | 0.00428151251496076 |
| CADM4 | 1946.45654683706 | 1714.81663180433 | 1903.28823092505 | 1234.54476067751 | 1522.98035699845 | 1558.84312277142 | 0.366374092412531 | 0.00445730063745739 |
| AC114488.1 | 93.6899516650451 | 166.621858960744 | 92.238564604497 | 66.0074057505103 | 57.7824525690469 | 73.6229434558291 | 0.835442807851302 | 0.00448187705828955 |
| CLGN | 45.8889559175731 | 58.5160099921661 | 42.647938473047 | 19.5959485821827 | 27.8593967743619 | 21.5960634137099 | 1.09091486634501 | 0.00463937187759912 |
| S100P | 30.5926372783821 | 20.827732370093 | 20.828062975209 | 11.345022863369 | 5.15914755080775 | 5.88983547646633 | 1.69297229853458 | 0.00467650793404749 |
| AC004706.3 | 104.206170729489 | 107.11405218905 | 55.541501267224 | 24.7527771564414 | 55.7187935487238 | 51.0452407960415 | 1.02033816496306 | 0.00475831405068 |
| ALDH3B1 | 632.885183696529 | 679.380793976844 | 646.661764754108 | 379.542583065434 | 563.378912548207 | 489.837983792783 | 0.451158611531006 | 0.00478117310811019 |
| FAM57B | 91.7779118351462 | 65.4585874488638 | 72.402314151917 | 30.9409714455517 | 46.4323279572698 | 46.1370445656529 | 0.894518554494003 | 0.00481139597204249 |
| BEST1 | 43.9769160876742 | 73.3929616850897 | 46.615188563563 | 19.5959485821827 | 25.7957377540388 | 31.4124558744871 | 1.09188873954828 | 0.00484682063760099 |
| PCK2 | 1881.4471926205 | 2881.16964452954 | 1714.84385162554 | 1672.8751894895 | 1509.56657336635 | 1436.13821701171 | 0.487979128416026 | 0.00502000484375243 |
| SARS | 3746.64204668686 | 4438.29058838887 | 3639.95195804843 | 3501.4866019216 | 2835.46749392394 | 2849.69873136362 | 0.364240983917718 | 0.00503410846951135 |
| H2AFY2 | 523.898913392293 | 520.693309252326 | 571.284013034304 | 372.323023061472 | 436.463882798336 | 419.159958075187 | 0.395913248668952 | 0.00512788453461325 |
| PACSIN3 | 1706.49554818475 | 1220.90183559926 | 1544.25209773335 | 1024.14615484776 | 1135.01246117771 | 1216.2510258903 | 0.405743015617697 | 0.00517054542867085 |
| FGF21 | 16.2523385541405 | 30.7457001653754 | 6.942687658 | 2.06273142970345 | 6.19097706096931 | 2.94491773823316 | 2.27020240352479 | 0.00521473075360749 |
| CORO6 | 1094.64280261711 | 1065.18974121333 | 1266.54459139723 | 813.74754901801 | 917.296434533619 | 939.428758496379 | 0.359413797962828 | 0.00535188403205934 |
| PRIM1 | 507.646574838153 | 484.988625189309 | 479.045448429807 | 334.162491611959 | 387.967895820743 | 393.637337677166 | 0.399267772756478 | 0.00558921777969031 |
| AL118558.3 | 38.2407965979776 | 37.6882776220731 | 25.787125588354 | 14.4391200079241 | 13.4137836321002 | 13.7429494450881 | 1.29060338771186 | 0.00564832068568712 |
| WASH7P | 351.815328701394 | 255.883569118286 | 280.682943904007 | 156.767588657462 | 266.21201362168 | 142.33769068127 | 0.653165121579205 | 0.00567821322911867 |

**Supplementary Table S3:** The top100 differentially expressed genes (Down).

| **Gene Name** | **OE_1** | **OE_2** | **OE_3** | **OENC_1** | **OENC_2** | **OENC_3** | **log2FoldChange** | ***p*-value** |
| --- | --- | --- | --- | --- | --- | --- | --- | --- |
| SLC16A2 | 842.253545070457 | 1238.75417763077 | 885.688582707697 | 1805.92136670537 | 1985.23997755082 | 2006.47061898286 | -0.966787209 | 2.24241816084919e-10 |
| SERPINA1 | 5155.81540132233 | 6061.8619164766 | 5487.69868770626 | 8808.89457054857 | 8598.2353081762 | 8850.45944263673 | -0.652454764 | 5.0020316873457e-10 |
| IL32 | 552.579510840776 | 686.323371433542 | 659.555327548285 | 1009.70703483984 | 1221.68614003128 | 1089.61956314627 | -0.807011823 | 6.03680934007722e-09 |
| PERP | 5845.10576000088 | 7992.89024621808 | 5837.8085081943 | 11172.7847889887 | 11298.533136269 | 10559.493370058 | -0.747404739 | 3.04286393640112e-08 |
| MPZL2 | 5258.10953222192 | 6857.28293365825 | 5338.92680931191 | 9859.85623398248 | 8944.93002359049 | 9027.15450693072 | -0.673176062 | 1.1072965041526e-07 |
| ETS1 | 2777.23785292812 | 3554.59965782921 | 2785.00956354223 | 4993.87279131205 | 4578.2275365868 | 4306.45137254296 | -0.606260803 | 2.65405018355009e-06 |
| WNT5A | 2564.0454118944 | 3295.74069837234 | 2700.70549911877 | 4677.24351685257 | 4287.25161472124 | 3989.38189605986 | -0.597634313 | 2.81422508851592e-06 |
| TMEM132A | 1965.57694513605 | 2149.2236212377 | 2027.26479625368 | 2686.70768718874 | 2887.05896943202 | 3041.11838434878 | -0.488204953 | 7.13772655564766e-06 |
| BACE2 | 3212.22691423012 | 3393.92857954564 | 3358.27720162179 | 4339.98692809605 | 4604.02327434084 | 4539.09987386338 | -0.43632514 | 1.05918615462685e-05 |
| CPA4 | 82.2177126856518 | 124.966394220558 | 120.009315238109 | 181.520365813903 | 238.352616847318 | 215.960634137099 | -0.959582063 | 1.86700397577494e-05 |
| ZMIZ2 | 5189.27609834556 | 5572.90610416918 | 5119.7362418109 | 6790.51186658375 | 7265.11158104748 | 7110.99469858701 | -0.414418229 | 3.1417211810715e-05 |
| JAG1 | 816.441007366822 | 1004.69013766211 | 873.786832436149 | 1323.24221215476 | 1336.21921565921 | 1218.21430438245 | -0.525005945 | 3.51802941190482e-05 |
| AL161431.1 | 1012.42508993146 | 1068.16513155191 | 1062.23121173566 | 1525.3898922657 | 1417.73374696197 | 1394.90936867644 | -0.464975937 | 3.67107029787075e-05 |
| MMP9 | 34.4167169381798 | 53.557026094525 | 44.631563518305 | 87.6660857623965 | 93.8964854247011 | 122.704905759715 | -1.200328214 | 4.93425311515834e-05 |
| CXCR4 | 716.058916297131 | 743.84758464618 | 756.752954765927 | 1037.55390914083 | 1027.7021921209 | 1005.19858798359 | -0.470094151 | 5.35733432138812e-05 |
| SLC5A3 | 8451.21604815305 | 9196.93153656537 | 8489.91519370424 | 13474.7930645378 | 11975.413294935 | 10581.0894334718 | -0.463080382 | 7.68311238183101e-05 |
| MALT1 | 1167.30031615327 | 1100.89442527635 | 1206.04402751686 | 2042.10411540641 | 1493.05730120376 | 1558.84312277142 | -0.552014812 | 8.84403726703411e-05 |
| PPIF | 2875.70790416792 | 2522.13921034031 | 2890.14169094091 | 4691.68263686049 | 3417.41933765506 | 3846.06256613251 | -0.528505347 | 8.92131466100876e-05 |
| TRIB1 | 3082.20820579699 | 2956.54619977368 | 3333.48188855607 | 5333.19211149826 | 4032.38972571134 | 4060.05992177745 | -0.518492945 | 9.41969999914527e-05 |
| SOD2 | 1650.09037320273 | 2001.44590108799 | 1716.8274766708 | 3640.72097342658 | 2318.520909 | 2450.17155820999 | -0.647506708 | 9.42673953050125e-05 |
| FN1 | 2926.37695966024 | 3613.11566782138 | 2850.46919003575 | 4171.87431657522 | 4688.63329417409 | 4273.07563817632 | -0.484089354 | 9.78965718486202e-05 |
| CTSS | 237.092938907461 | 419.530037740445 | 226.133255159412 | 581.690263176372 | 484.959869775929 | 499.65437625356 | -0.827552263 | 0.000120085458038882 |
| MT-ND1 | 32869.8767157916 | 28123.3894803028 | 29721.6458656232 | 46639.3889913098 | 38265.3973843411 | 39146.7914943334 | -0.451520521 | 0.000122113792681254 |
| BIRC3 | 832.693345920962 | 1513.48188556009 | 786.507330444797 | 1811.07819527963 | 1909.91642330903 | 1642.28245868803 | -0.775791552 | 0.000123468338285123 |
| TAPBP | 1825.99803755343 | 2267.24743800156 | 1911.22273110608 | 2999.21149878881 | 2709.58429368423 | 2608.2154768285 | -0.470050262 | 0.000149515664029797 |
| BIRC2 | 1290.62688518174 | 1375.62213320567 | 1200.09315238109 | 2253.53408695102 | 1632.35428507557 | 1722.7768768664 | -0.53661895 | 0.000168913372202441 |
| CPEB2 | 130.018708433124 | 160.671078283575 | 136.870128122802 | 285.688303013927 | 232.161639786349 | 205.162602430244 | -0.757714509 | 0.000181038023656164 |
| TGFBI | 6914.8920448293 | 6959.43800194966 | 6811.76840541597 | 8971.85035349515 | 8709.67289527365 | 8642.35192246826 | -0.347702759 | 0.000203427046675249 |
| ABAT | 685.466279018748 | 1153.45965459134 | 622.858264211012 | 1232.48202924781 | 1467.26156344973 | 1343.8641278804 | -0.716061204 | 0.000243504687070529 |
| SRSF1 | 1329.82370169467 | 1207.01668068587 | 1356.79953095647 | 2194.74624120447 | 1669.50014744139 | 1665.84180059389 | -0.506068758 | 0.00024803799556697 |
| LTA | 21.9884580438371 | 32.7292937244319 | 18.844437929951 | 57.7564800316965 | 66.0370886503393 | 59.879994010741 | -1.320683526 | 0.000273547204370624 |
| ID2 | 1624.2778354991 | 1541.25219538688 | 1501.60415926031 | 2110.17425258663 | 2057.46804326213 | 1954.44373894074 | -0.391368443 | 0.000276328576776986 |
| EIF4BP3 | 69.7894537913091 | 67.4421810079203 | 77.361376765062 | 282.594205869372 | 103.182951016155 | 114.851791791093 | -1.221637988 | 0.000279656925071382 |
| NEURL3 | 33.4606970232304 | 65.4585874488638 | 38.680688382531 | 88.6974514772482 | 99.0556329755089 | 110.925234806782 | -1.11990865 | 0.000300034537615659 |
| DAP | 4580.29141252277 | 4889.55812307422 | 5129.65436703719 | 7795.06207284933 | 6021.75702130281 | 6021.37513544074 | -0.442351829 | 0.00054154568826066 |
| MRPS6 | 3319.30114470446 | 2904.97276723821 | 3292.81757512828 | 4281.19908234951 | 4175.8140276238 | 3863.73207256191 | -0.3724278 | 0.000671080799878597 |
| LDLRAD3 | 806.880808217327 | 821.207733449383 | 886.680395230326 | 1180.91374350522 | 1051.43427085462 | 1103.36251259136 | -0.407626238 | 0.000702111161891947 |
| C3 | 263.861496526045 | 473.08706383497 | 242.994068044105 | 527.027880289231 | 585.047332261599 | 519.287161175115 | -0.735540827 | 0.000713795942471736 |
| AL450405.1 | 1141.48777844963 | 1261.56550355992 | 873.786832436149 | 1620.27553803206 | 1498.21644875457 | 1484.23854006951 | -0.49010479 | 0.000734589030200065 |
| TNFAIP3 | 242.829058397158 | 482.013234850725 | 243.985880566734 | 521.871051714972 | 628.384171688385 | 510.452407960415 | -0.777734658 | 0.000745034882178925 |
| SGK1 | 184.511843585242 | 219.187088275741 | 263.822131019314 | 339.319320186217 | 341.535567863473 | 327.867508189959 | -0.596132317 | 0.000751228852348351 |
| ARHGAP31 | 324.09075116786 | 402.669492488465 | 342.175320307005 | 487.835983124865 | 500.437312428352 | 523.213718159425 | -0.500180771 | 0.0007586425554248 |
| GSK3B | 1023.89732891085 | 1054.27997663852 | 1015.6160231721 | 1448.03746365182 | 1323.83726153727 | 1273.1861021628 | -0.386693235 | 0.000817408235189175 |
| MAP4K4 | 2652.9552639847 | 3141.02040076594 | 2749.30431272759 | 3843.90001925237 | 3676.40854470561 | 3543.71767834057 | -0.373032023 | 0.000852126591457348 |
| FAT1 | 3943.58214916644 | 4945.0987427278 | 3673.67358381782 | 6163.4415119539 | 5639.98010254304 | 5236.06373857856 | -0.439767839 | 0.000938063950429996 |
| GLI3 | 5369.963862 | 6031.11621631123 | 5473.81331238945 | 7321.66520973239 | 7331.14866969782 | 6757.60456999903 | -0.343429413 | 0.000954501041823239 |
| TMEM123 | 7648.15931959552 | 9723.57562649486 | 7911.68849301153 | 11947.3404408424 | 11145.8223687651 | 10396.5412552091 | -0.405529701 | 0.000957050613956412 |
| CSF2 | 391.96816512927 | 517.717918913741 | 379.864196166907 | 522.902417429824 | 650.052591401777 | 745.06418777299 | -0.573189527 | 0.00108186472364994 |
| ZC3H12C | 432.121001557147 | 355.06324707111 | 384.823258780052 | 725.050097540762 | 494.246335367383 | 520.268800421192 | -0.569340674 | 0.00109338536678873 |
| DOCK9 | 6203.61322810692 | 6808.68489146137 | 6920.86778290516 | 10377.601822838 | 8069.93859897349 | 8028.82739366968 | -0.409528296 | 0.00122830182041979 |
| SREK1 | 1153.91603734397 | 1251.64753576464 | 1183.2323394964 | 1748.16488667367 | 1515.75755042732 | 1443.99133098033 | -0.391520977 | 0.00130923957956174 |
| ROBO4 | 87.9538321753485 | 201.334746244233 | 86.287689468723 | 226.900457267379 | 246.607252928611 | 227.740305090031 | -0.90146799 | 0.0013447453787203 |
| PRDM1 | 239.00497873736 | 268.776927252153 | 233.075942817815 | 397.075800217914 | 396.222531902036 | 298.418330807627 | -0.558872369 | 0.00137061277643903 |
| MGAT4B | 4777.23151500235 | 4972.86905255459 | 5123.70349190141 | 6274.82900915789 | 6064.06203121944 | 6097.9429966348 | -0.309831584 | 0.00138449230642016 |
| MAP3K8 | 288.718014314731 | 334.235514701017 | 297.5437568 | 383.668045924841 | 474.641574674313 | 444.682578473208 | -0.501598574 | 0.00140066210307575 |
| DDX3X | 6151.98815269965 | 6044.00957444509 | 6150.22945282243 | 9243.09953650115 | 7071.12763313711 | 7636.17169523859 | -0.384552632 | 0.00144289556007092 |
| B2M | 4896.73400437103 | 5052.21279491685 | 5211.9748064154 | 7011.22412956202 | 6231.21841186561 | 5965.42169841431 | -0.341321727 | 0.00145981622500342 |
| MT2A | 6446.44228650407 | 6655.94818741402 | 5417.2799985996 | 9465.87453090912 | 7481.79577818141 | 7683.29037905032 | -0.411389278 | 0.00155726230186065 |
| CST3 | 7086.01960960525 | 7576.33559881622 | 7397.92960628971 | 8541.77085040198 | 9744.59789396569 | 9293.17874261779 | -0.322162217 | 0.00166648863051001 |
| SLC22A4 | 262.905476611096 | 236.047633527721 | 278.699318858749 | 418.7344802 | 337.408249822827 | 356.335046326213 | -0.516402624 | 0.00173578321366618 |
| ANO1 | 5675.89023505483 | 5880.36310582293 | 6065.92538839896 | 8178.73011877417 | 6987.54944281402 | 7080.5638819586 | -0.336202989 | 0.00184130802764689 |
| SLC12A2 | 598.468466758349 | 654.585874488638 | 624.84188925627 | 1086.02809773886 | 760.458348989063 | 783.348118370021 | -0.485759106 | 0.00203763109044255 |
| SIK2 | 2506.68421699743 | 2625.28607541125 | 2615.40962217267 | 3399.38139615128 | 3154.30281256386 | 3098.05346062129 | -0.317069781 | 0.00212291304546783 |
| CD44 | 4456.96484349429 | 4255.79998095568 | 4545.47679120871 | 7033.91417528876 | 5147.79742619598 | 5382.32798624414 | -0.405706881 | 0.00215249301185406 |
| PSTPIP2 | 357.551448191091 | 434.406989433369 | 406.64313427789 | 598.1921146 | 557.187935487238 | 490.819623038861 | -0.457771808 | 0.00229939999988949 |
| MTATP6P1 | 4783.923654 | 3920.57266947513 | 4782.51998411704 | 9468.96862805368 | 5471.79189238671 | 5205.63292195015 | -0.578910608 | 0.00232148924876186 |
| CCNL1 | 1850.85455534212 | 2245.42790885193 | 2031.23204634419 | 2956.92550447989 | 2547.58706058887 | 2462.932868 | -0.378827258 | 0.00236451737180137 |
| PANX1 | 625.237024376934 | 691.282355331183 | 664.51439016143 | 1080.87126916461 | 781.094939192294 | 839.301555396452 | -0.447357103 | 0.00273115813656771 |
| PLA2G4C | 85.0857724305002 | 120.007410322917 | 80.336814332949 | 157.798954372314 | 147.551619953102 | 156.080640126358 | -0.693513689 | 0.00281524402832032 |
| AC099560.2 | 42.0648762577754 | 33.7210905039601 | 31.738000724128 | 130.983445786169 | 55.7187935487238 | 53.0085192881969 | -1.154913377 | 0.00282367950305928 |
| PRRC2C | 3198.84263542083 | 3290.7817144747 | 3302.73570035457 | 4716.43541401693 | 3836.34211878065 | 3904.96092089717 | -0.347289626 | 0.00282858979882144 |
| MT-CO3 | 103286.479571308 | 90698.823691078 | 98319.3671807354 | 118955.658819568 | 122621.587158089 | 117087.965993658 | -0.295162923 | 0.0028476944128495 |
| PPP4R1 | 1113.7632009161 | 1208.0084774654 | 1164.38790156645 | 1694.53386950138 | 1383.68337312664 | 1430.24838153524 | -0.370984356 | 0.00286087107692378 |
| RPS2P46 | 736.135334511069 | 721.03625871703 | 949.164584155953 | 1426.37878363993 | 1028.73402163107 | 962.006461156167 | -0.505870269 | 0.00305834238404336 |
| CLOCK | 685.466279018748 | 645.659703472884 | 729.974016654944 | 1193.29013208344 | 812.049824497141 | 857.952701071928 | -0.474127175 | 0.00306700083158926 |
| CRYM-AS1 | 9.5601991494944 | 24.794919488206 | 11.901750271548 | 38.1605314495138 | 40.2413508963005 | 41.2288483352643 | -1.373432272 | 0.00317370519197749 |
| CWC22 | 486.614136709265 | 522.676902811382 | 461.192823022485 | 751.865606126907 | 606.715751974992 | 613.524528798576 | -0.423300667 | 0.00341554037190373 |
| FCGBP | 32.504677108281 | 70.417571346505 | 45.623376040934 | 73.2269657544724 | 119.69222317874 | 96.2006461156167 | -0.962195422 | 0.00348739312506203 |
| PCSK6 | 668.257920549658 | 754.75734922099 | 658.563515025656 | 885.943149057631 | 869.832277066188 | 922.740891313058 | -0.363917765 | 0.00359096224753004 |
| EIF5AL1 | 278.201795250287 | 257.867162677342 | 280.682943904007 | 449.675451675352 | 328.121784231373 | 371.059635017379 | -0.49207121 | 0.00360172358793856 |
| PRICKLE3 | 600.380506588248 | 625.823767882319 | 651.620827367253 | 817.873011877417 | 812.049824497141 | 775.4950044 | -0.357235151 | 0.00366897667875653 |
| IL6ST | 1514.33554527991 | 1975.65918482025 | 1540.28484764284 | 3075.53256168784 | 2048.18157767068 | 1990.76439104562 | -0.50009054 | 0.00373211017234549 |
| SGPL1 | 2421.59844456693 | 3309.62585328574 | 2559.86812090545 | 3609.78000198103 | 3691.88598735803 | 3514.26850095824 | -0.383571622 | 0.00380024604890568 |
| SHH | 1072.65434457327 | 1521.41625979632 | 1013.63239812684 | 1554.26813228155 | 1644.73623919751 | 1725.72179460463 | -0.4490941 | 0.00390938872944481 |
| NFAT5 | 2143.39664931664 | 2754.21965674992 | 2342.6611784497 | 3569.55673910182 | 3065.56547468997 | 2831.04758568815 | -0.38673709 | 0.00400459535613442 |
| FOXQ1 | 3722.74154881312 | 3693.45120696317 | 3527.87714299135 | 4322.45371094357 | 4492.58568724339 | 4491.98119005165 | -0.282034193 | 0.00400900565004327 |
| CSF1 | 76.4815931959552 | 126.949987779615 | 92.238564604497 | 136.140274360428 | 176.442846237625 | 167.86031107929 | -0.701426489 | 0.00406535074094477 |
| SLC25A13 | 1119.49932040579 | 1131.64012544172 | 1177.28146436062 | 1638.84012089939 | 1360.98312390309 | 1376.25822300097 | -0.352038297 | 0.00408859189247555 |
| CD83 | 202.676221969281 | 270.760520811209 | 227.125067682041 | 369.228925916917 | 277.562138233457 | 365.169799540912 | -0.531200606 | 0.00418653340069825 |
| URB2 | 560.227670160372 | 476.062454173555 | 589.136638441626 | 927.1977777 | 635.606978259515 | 695.982225469104 | -0.474556298 | 0.00430067220378278 |
| PIK3R1 | 543.019311691282 | 546.48002552006 | 503.840761495532 | 860.159006186338 | 632.511489729031 | 660.643212610306 | -0.434302254 | 0.0043102077224316 |
| GOLGA7B | 670.169960379557 | 877.740149882492 | 668.481640251946 | 877.692223338817 | 1130.88514313706 | 984.584163815954 | -0.433564839 | 0.00440267240007279 |
| AC007485.2 | 49.7130355773709 | 55.5406196535814 | 60.500563880369 | 102.105205770321 | 101.119291995832 | 80.4944181783731 | -0.775207683 | 0.00443363787787818 |
| TACSTD2 | 1240.91384960437 | 1666.21858960744 | 1321.09428014183 | 1951.34393249946 | 1804.66981327255 | 1764.98736444774 | -0.384946881 | 0.00445049226772086 |
| CDK2AP1 | 748.563593405411 | 625.823767882319 | 751.793892152782 | 934.417337655662 | 886.341549228772 | 923.722530559136 | -0.368179192 | 0.00445157368268078 |
| GJB2 | 846.077624730254 | 948.157721228997 | 961.066334427501 | 1595.52276087562 | 1040.08414624284 | 1148.51791791093 | -0.45773826 | 0.00457604215343794 |
| TNFRSF11B | 402.484384193714 | 516.726122134213 | 350.109820488037 | 507.431931707048 | 686.166624257431 | 598.79994010741 | -0.497923951 | 0.00478696725522206 |
| CD74 | 472.273837985023 | 585.160099921661 | 497.889886359758 | 610.56850319222 | 787.285916253263 | 690.092389992638 | -0.425027244 | 0.00488623995528089 |
| TGFBR3 | 148.183086817163 | 119.015613543389 | 140.837378213318 | 195.959485821828 | 204.302243011987 | 195.346209969467 | -0.545189297 | 0.00501869009156009 |
| RBM25 | 2963.66173634326 | 2984.31650960047 | 3136.1111965529 | 4123.40012797719 | 3510.2839935696 | 3617.3406217964 | -0.308630898 | 0.00502205861837678 |
